# Bromalites from the Upper Triassic Polzberg section (Austria); insights into trophic interactions and food chains of the Polzberg palaeobiota

**DOI:** 10.1038/s41598-020-77017-x

**Published:** 2020-11-25

**Authors:** Alexander Lukeneder, Dawid Surmik, Przemysław Gorzelak, Robert Niedźwiedzki, Tomasz Brachaniec, Mariusz A. Salamon

**Affiliations:** 1 Natural History Museum Vienna, Burgring 7, 1010 Vienna, Austria; 2 Faculty of Natural Sciences, University of Silesia in Katowice, Będzińska 60, 41-200 Sosnowiec, Poland; 3 Institute of Paleobiology, Polish Academy of Sciences, Twarda 51/55, 00-818 Warszawa, Poland; 4 Institute of Geological Sciences, Wrocław University, Borna 9, 50-204 Wrocław, Poland

**Keywords:** Evolution, Zoology, Ecology, Environmental sciences

## Abstract

A rich assemblage of various types of bromalites from the lower Carnian “Konservat-Lagerstätte” from the Reingraben Shales in Polzberg (Northern Calcareous Alps, Lower Austria) is described for the first time in detail. They comprise large regurgitalites consisting of numerous entire shells of ammonoid *Austrotrachyceras* or their fragments and rare teuthid arm hooks, and buccal cartilage of *Phragmoteuthis*. Small coprolites composed mainly of fish remains were also found. The size, shape and co-occurrence with vertebrate skeletal remains imply that regurgitalites were likely produced by large durophagous fish (most likely by cartilaginous fish *Acrodus*). Coprolites, in turn, were likely produced by medium-sized piscivorous actinopterygians. Our findings are consistent with other lines of evidence suggesting that durophagous predation has been intense during the Triassic and that the so-called Mesozoic marine revolution has already started in the early Mesozoic.

## Introduction

**Figure 1:**
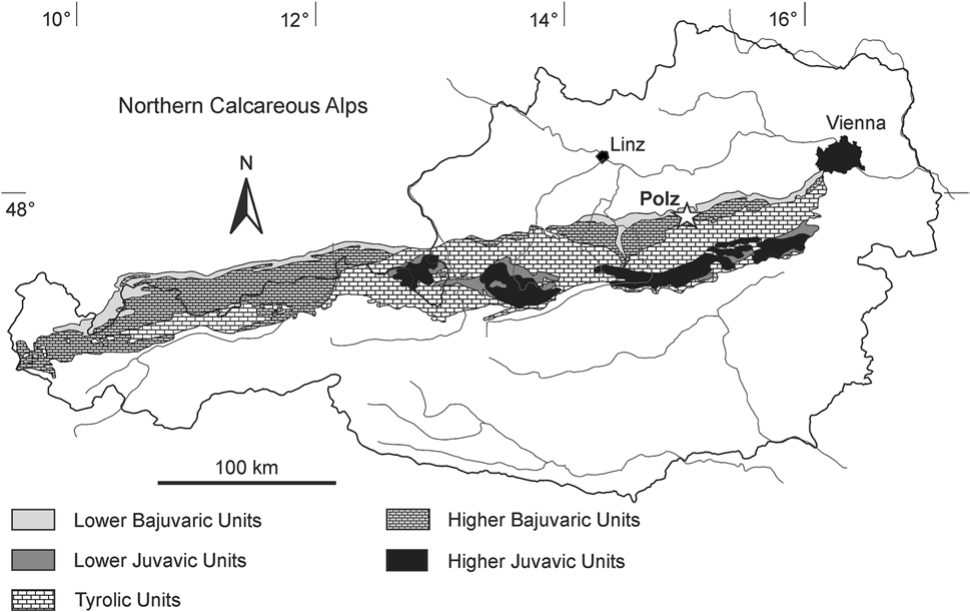
Map of Austria and the Northern Calcareous Alps with their main tectonic subdivisions (Bajuvaric, Tyrolic and Juvavic Units). Indicated with asterisk is the position of the Polzberg locality (Polz). Prepared by AL using CorelDRAW X7; www.coreldraw.com.

Bromalites, the fossilized products of digestion, are precious source of palaeobiological information^1–4^. They provide unique insights into dietary habits^5^, trophic interaction between animals^5–7^, health condition (e.g., parasitic infections system^8^), and some physiological aspects of extinct vertebrates^9^. The Triassic bromalites were recently described from a number of sites comprising localities in Germanic Basin epicontinental sea^5,8,10^. Until recently, a detailed study on bromalites from Northern Calcareous Alps has been lacking. They have been only briefly mentioned by Glaessner^11^ from the Upper Triassic Polzberg locality (the Northern Calcareous Alps). This locality (also known as Pölzberg^12^; southern part of the Lunz Nappe, Lower Austria; Fig. 1) comprises the lower Carnian Reingraben Shales which form at this locality a fossiliferous site known as “Konservat Lagerstätte” sensu Seilacher^13^ (see also^14,15^). Lithologies and the fossil content of the area, and distinct parts of the Polzberg section are known since the nineteenth century^12,16^. More recently^15,17–20^ new lithological and palaeontological data for the Polzberg locality were provided. During the last decade, private collectors detected new important biostratinomic elements within distinct layers of the thinly laminated deposits of the Reingraben Shales from Polzberg.

In this paper, we report fossilized vomits (regurgitalites) and coprolites, which provide new insights into pelagic invertebrates-vertebrates trophic chain of the Late Triassic Polzberg biota.

## Geologic setting

The Polzberg outcrop (710 m above sea level) is located on the western slope of Mount Schindelberg (1066 m), 4 km northeast of Lunz am See around Polzberg (postal code 3293) in the Lunz Nappe of Lower Austria. Hidden in a small mountain creek, the locality is accessible from the south over the Zellerrain street 71 via Mariazell or from the west over Lunz am See the Weißenbach street 25 and then street 71 (1:50,000, sheet 58 Baden 1996^21^; Fig. 1). The exact position of the fossiliferous locality (Reingraben Shales) was determined by GPS (global positioning system): N 47°53′4.98″ and E 15°4′28.15″.

Excavation campaigns were organized by the Austrian Geological Survey in 1885 and 1909. During these years, two fossil mines were driven into the basal part of the Reingraben Shales yielding better-preserved fossils, not harmed by weathering of soft marly deposits on the surface. The historical abandoned and collapsed mines were located approximately at N 47°53′23.31″ and E 15°4′45.80″.

Classically, the Northern Calcareous Alps (NCA) are subdivided from north to south into the Lower and Upper Bajuvaric Units, the Tyrolic Unit, and the Lower and Upper Juvavic Units (Fig. 1^22,23^). The northernmost tectonic elements of the NCA in Lower Austria are the Frankenfels Nappe, followed subsequently to the south by the Lunz Nappe. Within the Lunz Nappe in Lower Austria, the Reifling basin is located between the Polzberg and Großreifling.

The lower Upper Triassic (*Austrotrachyceras austriacum* Zone, Julian 2, lower Carnian) Polzberg section is located in the southern part of the Reifling basin^14^ on the easternmost part of the Geological map 1:50,000, sheet 71 Ybbsitz^24,25^ (Fig. 1). The Polzberg locality is located in the southern area of the Lunz Nappe (Upper Bajuvaric Unit of the Northern Calcareous Alps) that is bordered to the north by the Low-Bajuvaric Unit of the Frankenfels Nappe, and to the south of the lake Lunzer See by the Tyrolic Ötscher Nappe.

**Figure 2:**
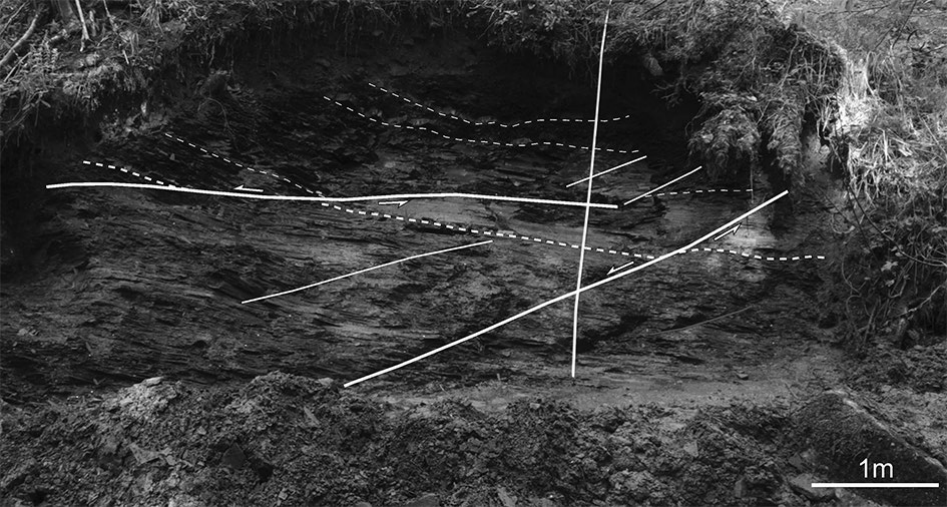
Section within the Carnian Reingraben shales at Polzberg in the Polzberg ravine, 2. Mai 2014 by G. Bryda. Solid lines = main fault zones with indicated direction of shearing, dashed lines = marker layers. Prepared by AL using CorelDRAW X7; www.coreldraw.com.

The known conservation Fossil-Lagerstätte in Reingraben Shales from Polzberg in Lower Austria is poorly understood^12^. Teller^16^ published first preliminary data on the Polzberg locality. The deposits consisting of millimetre-laminated, dark grey, brownish, slightly bituminous marls, with clearly visible bright/dark stratification without any bioturbation^14,18,19^ are included the Reingraben Member in the Lunz Formation. Nevertheless we consider the Reingraben Shales as informal lithostratigraphic unit (= Reingrabener Schiefer^26^; “Halobienschiefer” of^27,28^) given the more siliciclastic and fine-grained facies types (Fig. 2).

## Fossil content in the Reingraben Shales

In the late 19th and early twentieth centuries, hundreds of fossils have been collected from Polzberg locality^29^. The fauna was dominated by ammonites of the ceratitid species *Austrotrachyceras austriacum* with original aragonite shell, hundreds of fish remains, and arthropods. The ammonites derive from several distinct ammonite layers. Rare ammonites of the genera *Carnites*, *Sageceras* and *Simonyceras* also occur. Over 100 fragments of coleoid proostraca and phragmocones of *Phragmoteuthis bisinuata*^30^, showing arm hooks and buccal cartilage have been recovered from the Polzberg deposits. The Reingraben Shales contain common remains of fossil fishes^11,17,31–33^. The lungfish *Ceratodus sturi*^16^ was also found in deposits from Polzberg^26^. A single conodont cluster with abundant *Mosherella,* assigned to Triassic jawless fishes, was reported. Noteworthy, Forchielli & Pervesler^15^ re-described the arthropods consisting of dozens of thylacocephalan *Austriocaris*^11^ and noted the presence of other arthropods (including crustaceans with decapods and isopods) from the Polzberg locality. Thousands of shells of the benthic bivalves *Halobia rugosa* (1–3 mm length), dozens of gastropods, and rare brachiopods appear in the Polzberg collections housed at the Natural History Museum Vienna and the Geological Survey Vienna. The Reingraben Shales are mostly scarce or barren of microfossils. Ostracods (*Dicerobairdia*), foraminifera (*Trocholina*), and pelagic crinoids (*Osteocrinus*^26^) are locally common.

## Palaeoenvironment of the Reingraben Shales

The Reingraben Shales^28^ are interpreted as deposits of a relatively deep marine environment within intra-platform basin as inferred from the dominance of a nektonic fauna^15,34,35^. There is some confusion in the designation to either Lunz or Polzberg localities in official collections of the Natural History Museum Vienn, the Geological Survey Vienna or the University of Vienna. As stated by Forchielli and Pervesler^15^ the fauna of the Polzberg Lagerstätte were often referred to the Lunz Lagerstätte in the scientific past. Both localities are different in biostratigraphy, lithology, and depositional environments.

**Figure 3:**
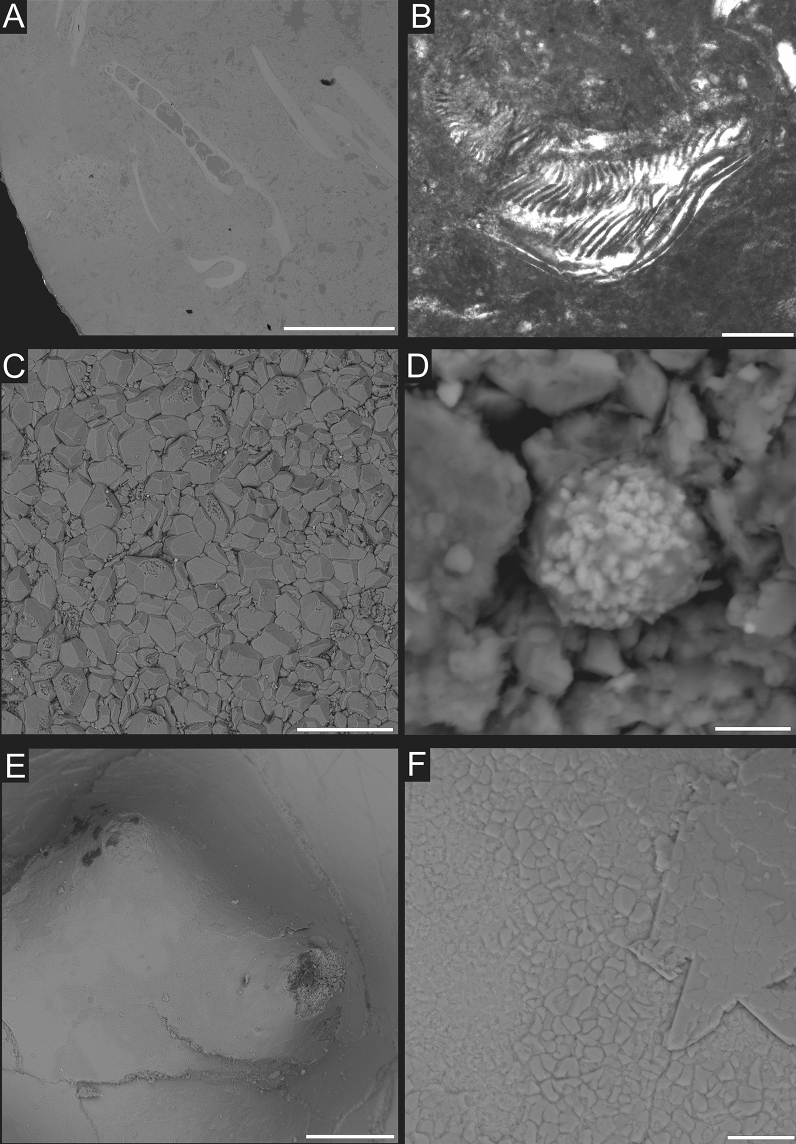
The detailed images of bromalites from Polzberg section. **(A)** SEM-image of specimen NHMW 2020/0033/0008 AS244 thin-section showing small fish jaw embebbed in coprolite matrix; **(B)** microscope image of NHMW 2020/0033/0008 AS244 thin-section exhibiting aptychus/cephalic cartilage; **(C)** NHMW 2020/0033/0002 1910A, higher magnification of cephalopod remains showing calcium carbonate crystals; **(D)** sample NHMW 2020/0033/0002 1910A, iron oxide pseudomorphosis after pyrite framboids; **(E)** SEM image of sample NHMW 2020/0033/0001 Polz, detailed view of two ammonite tubercles; **(F)** SEM image of sample NHMW 2020/0033/0001 Polz, areas of calcium carbonate dissolution. Scale bar equals 100 μm for (**A**,**B**,**E**); it is 20 μm for (**C**); 10 μm for (**F**), and 5 μm for (**D**).

Data from the outcrops in the Polzberg area show a nektonic dominated fauna with abundant fishes and cephalopods. The well preserved soft bodied fauna, the abundance of organic material in the sediment, the presence of common framboidal pyrite (e.g., Fig. 3D) crystals^15^, the absence of sessile organisms, and lack of bioturbation suggest dysoxic to anoxic bottom conditions during deposition of the Reingraben Shales. As noted by Griffith^17^ the Polzberg basin was mainly normal marine with ephemeral and limited freshwater input.

## Results

**Figure 4:**
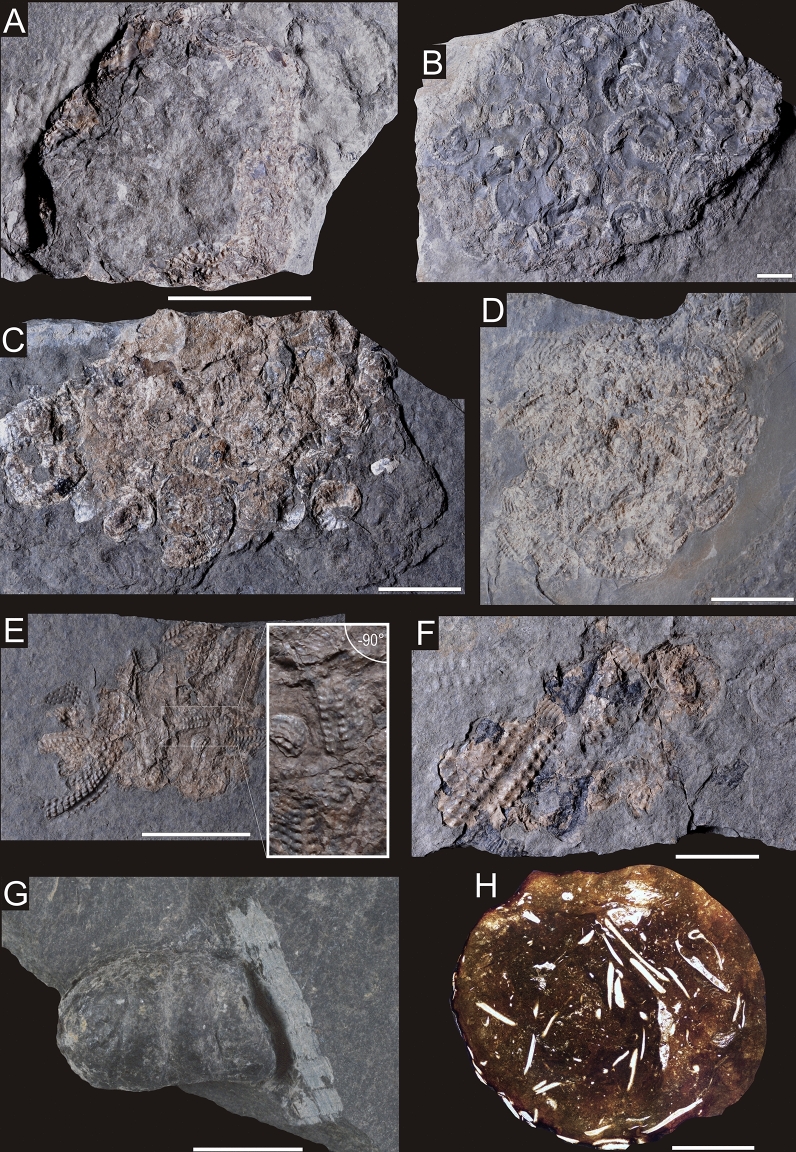
Bromalites from Polzberg section. **(A)** fragment of NHMW 2020/0033/0001 Polz specimen exhibiting ammonite hash and fragments of teuthid cartilage; **(B)** specimen NHMW 2020/0033/0002 1910A composed mainly of entire ammonite specimens; **(C)** specimen of NHMW 2020/0033/0003 AS93 exhibiting amonite hash and entire ammonites; **(D)** specimen NHMW 2020/0033/0004 AS186A composed of variously oriented ammonites; **(E)** specimen NHMW 2020/0033/0005 1910B showing fragmented ammonite specimens, dominated by external views; **(F)** specimen NHMW 2020/0033/0006 AS187 revealing fragmented ammonites, large specimen from external view with organix black material; **(G)** specimen NHMW 2020/0033/0007 AS193 showing entire coprolite; **(H)** thin-section of specimen NHMW 2020/0033/0008 AS244 exhibiting actinopterygian fish remains. Scale bar equals 10 mm.

**Figure 5:**
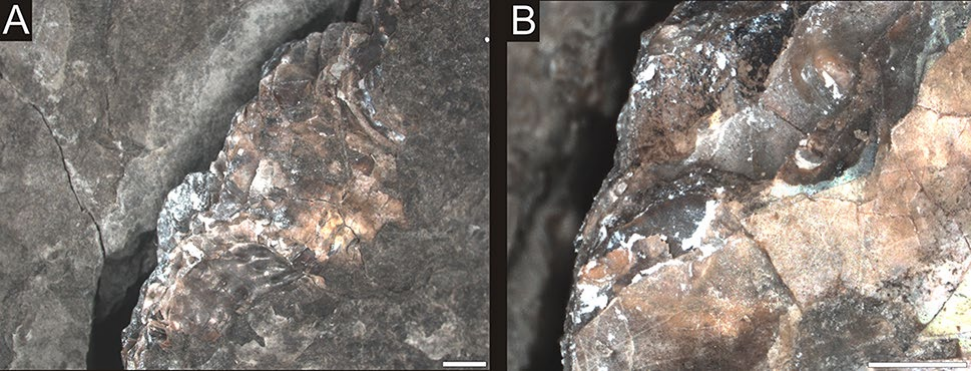
Enlargements of the angular shell fragments showing their jagged margins **(A**,**B)**. Both photographs taken from NHMW 2020/0033/0001 Polz. Scale bar equals 1 mm.
  - *NHMW 2020/0033/0002 1910 A* (slab): Massive, thick (< 10 mm), maximum dispersion 100 mm, composed of variously oriented entire ammonite specimens (with original white shell), ammonite hash with angular shell margins and teuthid cartilage fragments (Fig. 4B). Results of Raman spectroscopy show that skeletal debris on the surface of bromalites are composed of calcite (distinct peak at ~ 1090 cm^−1^); Supplementary Fig. S1). Closer examination under SEM indicates that these carbonate debris are crushed shell fragments of mollusk (Fig. 3C) and teuthid buccal cartilage (Supplementary Data S2).
  - *NHMW 2020/0033/0003 AS93* (slab and counter-slab): Thin fragile mass, maximum 2.5 mm thick, maximum dispersion 38 mm, composed of variously oriented entire ammonites (with original white shell) and ammonite fragments displaying angular shell margins and teuthid arm hooks (n = 13) (Fig. 4C).
  - *NHMW 2020/0033/0004 AS186 A* (slab and counter-slab): Thin (< 2 mm) fragile mass, max. dispersion 68 mm, composed of variously oriented entire ammonites (with original white shell) and ammonite fragments displaying angular shell margins (Fig. 4D).
  - *NHMW 2020/0033/0005 1910 B* (slab and counter-slab): Thin (< 3.5 mm) fragile mass, maximum dispersion 70 mm, composed of variously oriented entire ammonites (with original white shell) and ammonite fragments displaying angular shell margins with teuthid arm hooks (n = 4) (Fig. 4E).
  - *NHMW 2020/0033/0006 AS187* (slab and counter-slab): Thin (< 2.5 mm) mass, maximum dispersion 46 mm, composed of variously oriented fragmented ammonite specimens (with original white shell) and their fragments displaying angular shell margins, and associated organic black ink material (Fig. 4F).
- **Type B** Elongated, cylindrical, small (< 15 mm); they contain loosely dispersed remains of the body macrofossils/microfossils.
  - *NHMW 2020/0033/0007 AS193*: Small elongated bromalite, up to 5 mm thick, 8 mm in breadth, and 13 mm in length, with rare fish scales (Fig. 4G). XRD analysis shows that the host rock surrounding bromalite contains mainly clay minerals, represented by illite–smectite mixed layer structure and chlorite, however small amount of mica and kaolinite are also detected (Supplementary Fig. S2; Supplementary Data S3). The XRD spectrum shows numerous strong reflections indicating the presence of quartz, sodium feldspars (albite) and carbonate minerals—calcite, with an admixture of dolomite. Calcite reflections are doubled, which indicates the occurrence of two generations of calcium carbonate, differing in structure, which in turn may be related to the occurrence of a more Mg-rich variety. For example, the main calcite d_10.4_ peak consists of two overlapped reflections—3.027 and 3.010 Å. The first peak, 3.027 Å, represents a purer calcite variety; the second peak indicates a Mg-rich generation. In the analyzed bromalite sample, some minerals found in the parent rock sample are also present (quartz, chlorite and illite), but they represent only a small admixture. The main minerals detected in the bromalite matrix are apatite and calcite. The obtained apatite reflections match with a high score the carbonate apatite reference patterns. For the Rietveld refinement the 01-073-9696 (ICDD, PDF4 +) carbonate-fluorapatite structure was used. The calculated structural parameters (lattice parameters) of apatite are a = 9.3202(5) Å and c = 6.9012(5) Å, refined in the P63/m (no. 176) space group. Semi-quantitative XRD calculations show that apatite is present in an amount of about 53 wt%. Calcite constitutes up to 42 wt% (Supplementary Data S3).
  - *NHMW 2020/0033/0008 AS244*: Small elongated bromalite, up to 3 mm thick, 6 mm in breadth, and 11 mm in length, with relatively abundant fish scales (Fig. 4H). It is characterized by dark grey matrix as visible in CT scans with radiologically dense filamentous shapes located randomly throughout the bromalite matrix in some cases reaching to the peripheries. The further observations of thin-sections under optical microscope revealed these objects are crushed and etched small vertebrate skeletal remains, especially actinopterygian fish scales (Fig. 4H). Interestingly, a small fish mandible was observed in the section (Fig. 3A). An individual fragment of aptychus/cephalic cartilage was also observed (Fig. 3B).

Two morphotypes of bromalites are distinguished:

- **Type A.** Flat, slightly oval, large (> 40 mm) and up to about 1 cm thick; they contain numerous and densely packed body macrofossils:
  - *NHMW 2020/0033/0001 Polz* (slab): Massive, thick (< 11 mm), maximum dispersion (i.e., diameter) 50 mm, composed of variously oriented entire ammonite specimens (with original white shells), ammonite hash with angular shell margins and teuthid cartilage fragments (Figs. 4A, 5), including teuthid hook. SEM observations show some slight traces of calcium carbonate dissolution (Fig. 3F, Supplementary Data S1) in some ammonite shell fragments, suggesting their partial digestion in the digestive tract.

## Discussion

### Types of bromalites

Possible explanation for the origins of distinct accumulations of shells and/or shell hash (morphotypes A and B) reported herein is that they represent abiotic structures produced by post-mortem transport by bottom currents or waves or that they are ichnofossils (e.g., infills of decapod burrows). However, the occurrence of densely packed ammonoid shells and their fragments in distinct oval spots, the rarity of these shells in the surrounding host rock, the lack of size sorting (shell size from below 1 mm up to 20 mm) and preferential orientation, low degree of shell roundness and abrasion, lack of ripple marks, and the fact that they do not penetrate deep into the sediment, all suggest that these accumulations were produced by predator activity (i.e., they represent bromalites).

Distinguishing between different types of bromalites is not always easy to draw. However, a number of diagnostic criteria have been recently proposed^36–40^. More specifically, regurgitalites are thin, and commonly composed of randomly grouped and intermingled angular skeletal fragments of different size, revealing low degree of roundness and distortion of individual crystal fibers at the shell edges. Furthermore, they usually lack a phosphatic matrix and may contain debris, which are not significantly affected by gastric etching (including soft body tissues). By contrast, coprolites typically are massive, usually thick, possess a more or less regular shape, and contain a phosphatic matrix and fossil inclusions revealing signs of digestion. The gastric residues (fossilized stomach contents, i.e., consumulites) may reveal similar features to regurgitalites, however they are usually thicker and are associated with the body fossil of the producer.

The evidence suggests that Polzberg locality preserves two types of bromalites with regurgitalites (morphotype A) being the most common. An interesting feature is the presence of uncrushed ammonoid shells within some regurgitalites (NHMW 2020/0033/0003 AS93 (Fig. 4C); NHMW 2020/0033/0002 1910 A (Fig. 4B). In other accumulations, crushed ammonite fragments predominate. The presence of uncrushed shells may result either from the fact that the predator swallowed the entire ammonoids or from the fact that while swallowing it crushed only the body chamber containing the soft tissues. In this case, phragmocone remains ingested undamaged. Accumulations of ammonoid shells consisting of phragmocones, interpreted as a result of predation, have been described in the literature^42^. However, among ammonoids from studied bromalites from Polzberg inner moulds are unknown, thus it is uncertain if these shells represent entire specimens or if they are phragmocone parts of ammonoids.

### Potential producers of bromalites

Based on the size of regurgitalites (up to 100 mm) and the fact that in some of them entire (uncrushed) ammonite shells (diameter up to 20 mm) can be also found, it seems that they were produced by large durophagous predators. Cephalopods and arthropods noted from Polzberg section appear to be too small to produce these bromalites. Furthermore, these invertebrates have a rather alkaline gastric pH, and thus they produce regurgitates commonly containing specific hardparts (e.g., aptychi of ammonites) without etching-related features^43^.

A rich inventory of Triassic ichthyofauna and lack of the reptile remains in the Polzberg section allow to search probable bromalite producers among predatory fishes.

There is evidence that Palaeozoic and Mesozoic shelled cephalopods have been preyed upon by sharks and actinopterygian fishes^41,43–45^. Noteworthy, Recent sharks and actinopterygian fishes are known to attack on *Nautilus*^46^. Interestingly, predation experiments on living durophagous fish (*Diodon*) revealed that the critical size of the prey, i.e., the size above which this fish is incapable of crushing a given prey species is about 12% of the fish length^38,47^. We thus hypothesize that studied regurgitalites at hand (dispersed over 7 cm and containing intact ammonite shells up to about 2 cm in diameter) might have been produced by a fish having at least several dozen cm in length.

Glaessner^11^ and Griffith^17^ noted the occurrence of 12 genera (representing 13 species and 5 taxa of actinopterygiids indeterminate at the genus level) with the cartilaginous fish *Acrodus*, the coelacanthid? *Coelacanthus,* dipnoan *Ceratodus sturi*^16^, and the actinopterygiids: *Elpistoichthys*, *Gigantopterus*, *Saurichthys, Thoracopterus, Habroichthys*, *Nannolepis*, *Peltopleurus*, *Phaidrosoma*, *Phloidophoretes* and *Polzbergia*; of these genera, the first seven are predatory^17^.

Griffith^17^ stated that the Upper Triassic ichtiofauna of Polzberg region is characterized by the large content of flying fish, which, according to this author, suggests a strong predation pressure in this marine ecosystem. Furthermore, 55% of the genera of marine fish known from Polzberg were predatory^17^. On the other hand, the specimens of predatory fish from the Reingraben Shales of Polzberg, in which the body size could have been reconstructed, usually reveal rather small body sizes (a few to 11 cm in length)^17^. The largest specimens of predatory fish described by Griffith^17^ belonged to *Saurichthys* (up to about 40 cm in length), palaeoniscids, probably belonging to the family Acrolepididae (20–25 cm in length), and *Gigantopterus telleri* (18.6 cm in length). Apart from these taxa, remains of large sarcopterygian fishes are known, namely dipnoan (lungfish) *Ceratodus sturi*, with the length up to about 1.5 m^16^ and coelacanth *Coelacanthus lunzensis*^16,48^). However, Mesozoic dipnoans were restricted to freshwater environments and their remains found in marine deposits are commonly interpreted as a result of post mortem transport from freshwater ecosystems and/or as a result of redeposition from older, non-marine sediments^49^. Thus, it is doubtful if *Ceratodus* was a true member of marine palaeobiota in the Polzberg area.

Among large (several dozen cm in length) marine predatory vertebrates occuring in Polzberg region, which might have produced regurgitalites desribed herein, are *Acrodus*, *Saurichthys* and eventually coelacanths. For instance, the reconstructed standard length of the Middle Triassic specimens of shark *Acrodus* from the so-called Grenzbitumenzone from Monte San Giorgio (Switzerland) is 1.8–2.5 m^50^, marine species of the Late Triassic *Saurichthys* may reach up to about 1.1 m (Upper Triassic of Alps near Salzburg^51^) to 1.5 m in length (Norian, Southern Calcareous Alps, see^55^ Lower Triassic of Idaho, USA^52^). Triassic marine coelacanths may reach above 60 cm (e.g., Middle Triassic, Switzerland^53^; Late Triassic, England^54^).

In particular, a typical durophagous dentition (crushing or grinding teeth) with blunt and broad teeth is observed in *Acrodus*^58,59^. The actinopterygian assemblage of Polzberg does not contain typial durophagous taxa^17,55^ although durophagy sensu lato (the ability to consumption of hard prey^56,57^) is possible with other dental types, especially when dealing with thin-shelled preys, such as small ammonites occuring in regurgitalites. A Late Triassic fish *Legnonotus*^55^ with peg-like teeth is also considered a durophagous. Likewise, hybodont or ctenacant sharks with relatively narrow and long cusp (tearing-type of dentition sensu Cappetta^58^) might have attacked ammonites^42,45^. Small, thin-shelled scaphites were noted in the stomach of Cretaceous plesiosauroids and pliosauroids, which displayed conical, quite long teeth with an acute, but rounded apex^60^. Furthermore, thin-shelled ammonites might have been preyed upon by marine reptiles with crunching teeth (robust with a blunt apices conical teeth)^60^. Triassic species of *Saurichthys* are characterized by monognathic heterodonty—the teeth in one jaw have different size and shape. They possessed two types of conical teeth: robust with approximately circular in section small teeth, and larger, slender teeth with acute apex^61,62^. Triassic coelacanths commonly possessed robust, conically pointed teeth, however they also had small rounded teeth on the parasphenoid^63^. On the other hand, shells of ammonoids which have been attacked by predators having conical teeth commonly reveal shell damages in the form of indentations or holes of various size and shape, occuring both in the body chamber and in the phragmocone^42,45^. Similar damages were not recorded in ammonoid shells from Polzberg. Given the above, we argue that a durophagous shark *Acrodus* was likely a producer of regurgitalites studied, although other durophagous predators (until now not found in the Upper Triassic deposit of Polzberg), which were present in the latest Ladinian and early Carnian in the other areas of the east part of Alpine domain, including durophagous (placodonts) and semi-durophagous (thallatosaurs) reptiles (recorded in a few outcrops in NE Italy^64,65^; Slovenia^66^; Hungary (Bakony)^64^, some hybodontid sharks (e.g., *Palaeobates*, *Asteracanthus*; see^64,67^ and some actinopterygian fishes (e.g., *Colobodus*^64,67^), cannot be fully excluded.

Regarding coprolites, given their longitudinal shape, small size and occurrence of the fish scales, we hypothesize that they were likely produced by medium-sized piscivorous actinopterygians (such as, common in the fish assemblage from Polzberg, *Elpistoichthys* and *Thoracopterus* or rarer *Gigantopterus*, acrolepids and *Saurichthys*). These coprolites do not reveal a spiral morphology, which is characteristic for coprolites produced by chondrichthyans, dipnoans and some actinopterygian fishes (e.g.^3,68^).

## Conclusions

Discovery of bromalites at Polzberg locality not only proves for the first time the presence of large predators in the Reifling basin and provides insights into trophic interactions, and food chains of this Late Triassic ecosystem, but also constitutes another important evidence confirming previous hypotheses that the so-called Mesozoic marine revolution (a time of increased escalatory adaptions to shell-crushing predation^69^) has already started in the early Mesozoic^70^. Not so long ago, it has been argued that marine durophagous predation was not intense during the Triassic^5^. For instance, McRoberts^71^ pointed out that durophagous predators displayed low abundances and limited distribution during the Triassic. More recent reports, however, highlighted that innovations for durophagy appeared in many Triassic invertebrates (e.g., sea urchins) and vertebrates (including Chondrichthyes (hybodontids), some ichthyopterygian^72^ and sauropterygian vertebrates (such as placodonts, pachypleurosaurs, some pistosaurs and nothosaurs)^70,73–77^, which might have affected the evolution of shallow-marine benthic comunities. Nonetheless, the more direct evidence of durophagous predation in Triassic marine communities still remains limited^5, 8^. Our findings thus constitute a new important evidence for durophagy in the Triassic, and together with other lines of indicators (i.e., predatory or defensive behaviors of predators and prey inferred from functional morphology, taphonomy and trace fossils)^5,8,70,73,78,79^ confirm recent hypotheses about the early timing of Mesozoic marine revolution.

## Material and methods

Among the several dozens of bromalite specimens obtained from the locality, 8 representative bromalites were selected for detailed investigation. These bromalites came from the ravin Schindelberggraben (Fig. 2) near Polzberg (= Polzberggraben in^14^; or given as Polzberg locality in numerous collections), between mount Föllbaumberg (1014 m) to the west and mount Schindelberg (1066 m) to the east. The investigated fossil material is housed in the collections of the Natural History Museum Vienna (NHMW) and the Geological Survey Vienna. The bromalite material was collected over the last 10 years by Birgitt and Karl Aschauer (both Waidhofen an der Ybbs, Lower Austria).

Bromalites recorded herein have been investigated with a number of different analytical tools.

### Scanning electron microscopy

The SEM images of internal inclusions from selected bromalites were taken using the Phenom XL SEM, PhenomWorld (ThermoFisher Scientific, Eindhoven, The Netherlands) equipped with an energy-dispersive X-ray spectroscopy (EDS) detector, installed in the Faculty of Natural Sciences, University of Silesia in Katowice in low-vacuum settings with accelerating voltage 15 kV. Composite images were collected using Phenom SEM software, then stitched and processed using Image Composite Editor by Microsoft Research.

### Microtomography

Virtual sections of a selected specimen were made in the Faculty X-ray Microtomography Laboratory at Faculty of Computer Science and Material Science, University of Silesia in Katowice, Chorzów, Poland using the General Electric Phoenix v|tome|x micro-CT equipment at 160 kV, 70μA and scanning time of 20 min. Projection images were captured using a 1000 × 2024 pxs scintillator/CCD with an exposure time of 250 ms and processed using Volume Graphics VGSTUDIO Max software and analysed using Volume Graphics myVGL viewer app and Fiji image processing package.

### Thin-sectioning

Thin sections from two specimens were made in the Grindery at the Faculty of Natural Sciences, University of Silesia in Katowice, Sosnowiec, Poland. Specimens were embedded in Araldite epoxy resin, sectioned, mounted on the microscope slides and polished with silicon carbide and aluminium oxide powders to about 30 μm thick.

### Mineralogical studies

One specimen was examined using X-ray diffraction and Raman spectroscopy to determine its mineralogical composition. The powdered bulk X-ray diffraction analysis of coprolite matrix was performed using PANalytical X'Pert PROMPD PW 3040/60 diffractometer at the Laboratory of X-ray Diffraction, Faculty of Natural Sciences, University of Silesia in Katowice, Sosnowiec. Raman spectroscopy was used for in-depth characterization of carbonate phase of skeletal remnants observed in coprolite matrix. The analysis was performed using WITec alpha300 confocal Raman microscope equipped with a laser (λ = 532 nm), coupled with a CCD camera and with an Olympus MPLAN objective.

XRD analyses were performed on powdered samples using a PANalytical X’Pert Pro MPD (multipurpose diffractometer) powered by a Philips PW3040/60 X-ray generator and fitted with a 1D silicon strip detector (X’Celerator). The measurements were performed using Co Kα-radiation with a wavelength of 0.1789010 nm, an acceleration voltage of 40 kV, a current of 40 mA, and with 0.02° 2θ step sizes between the angles of 5° and 90° 20 and a 200 s measurement time per step. The data obtained were processed using HighScore + software and the ICSD database and PDF4 + ICDD database. All XRD analyses were performed at the Faculty of Earth Sciences, University of Silesia in Katowice, Sosnowiec. The diffractometer was manufactured in the Almelo Malvern Panalytical B.V. factory (Holland).

## Supplementary information

Supplementary Information 1.

Supplementary Information 2.
