# Supplementary material for: Bromalites from the Upper Triassic Polzberg section (Austria); insights into trophic interactions and food chains of the Polzberg palaeobiota
Source: Sci Rep. 2020 Nov 25;10:20545. doi: 10.1038/s41598-020-77017-x (PMC7689505; doi:10.1038/s41598-020-77017-x)

Supplementary information for

Bromalites from the Upper Triassic Polzberg section (Austria); insights into trophic interactions and food chains of the Polzberg palaeobiota

Alexander Lukeneder[1], Dawid Surmik[2], Przemysław Gorzelak[3], Robert Niedźwiedzki[4], Tomasz Brachaniec[2], Mariusz A. Salamon[2*]

[1] *Natural History Museum Vienna, Burgring 7, 1010 Vienna, Austria*

[2] *University of Silesia in Katowice, Faculty of Natural Sciences, Będzińska 60, 41-200 Sosnowiec, Poland, paleo.crinoids@poczta.fm (MAS - corresponding author)*

[3] *Institute of Paleobiology, Polish Academy of Sciences, Twarda 51/55, 00-818 Warszawa, Poland (orcid.org/0000-0001-5706-1881)*

[4] *Institute of Geological Sciences, Wrocław University, Borna 9, 50-204 Wrocław, Poland*

Supplementary Data S1. SEM-EDS report for sample NHMW 2020/0033/0001 Polz.

Polz

Contains 2 images with a total of 7 analyses

01. Image 1

5 analyses: 5x spot

02. Image 2

2 analyses: 2x spot

Image 1

1. spot

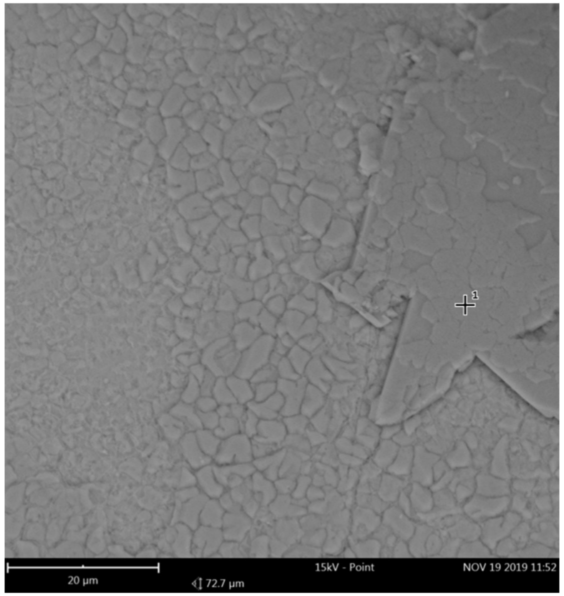

Element Symbol	Atomic Conc.	Weight Conc.	Oxide Symbol	Stoich. wt Conc.
O	79.57	62.48		
Ca	16.91	33.26	CaO	86.14
F	1.88	1.75	F	3.24
P	1.65	2.51	P_2O_5	10.63

FOV: 72.7 µm, Mode: 15kV - Point, Detector: BSD Full, Time: NOV 19 2019 11:52

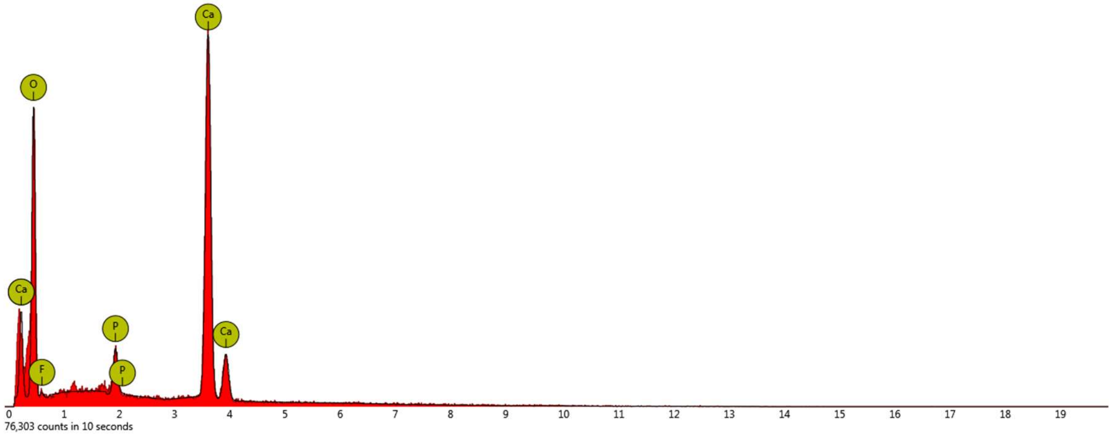

Disabled elements: Am, B, Br, C, Cs, Ho, In, Ir, N, Np, Os, Pm, Pu, Re, Sb, Sn, Tb, Tc, Te, Tm, W

2. spot

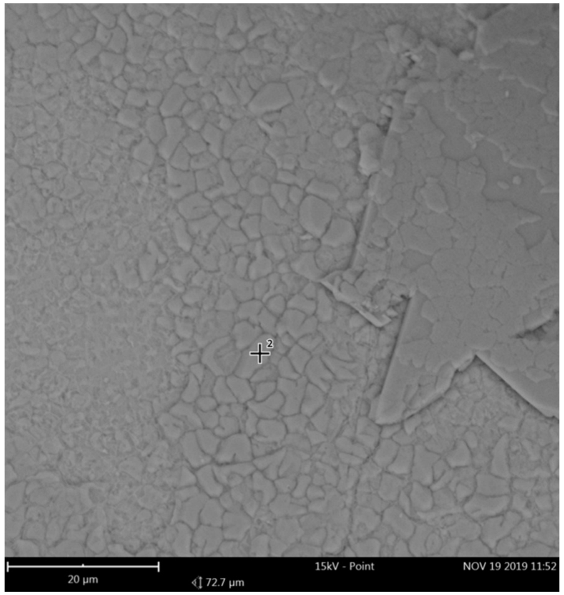

Element Symbol	Atomic Conc.	Weight Conc.	Oxide Symbol	Stoich. wt Conc.
O	77.74	59.37		
Ca	17.30	33.10	CaO	77.85
P	2.79	4.12	P_2O_5	15.86
F	1.73	1.57	F	2.64

FOV: 72.7 µm, Mode: 15kV - Point, Detector: BSD Full, Time: NOV 19 2019 11:52

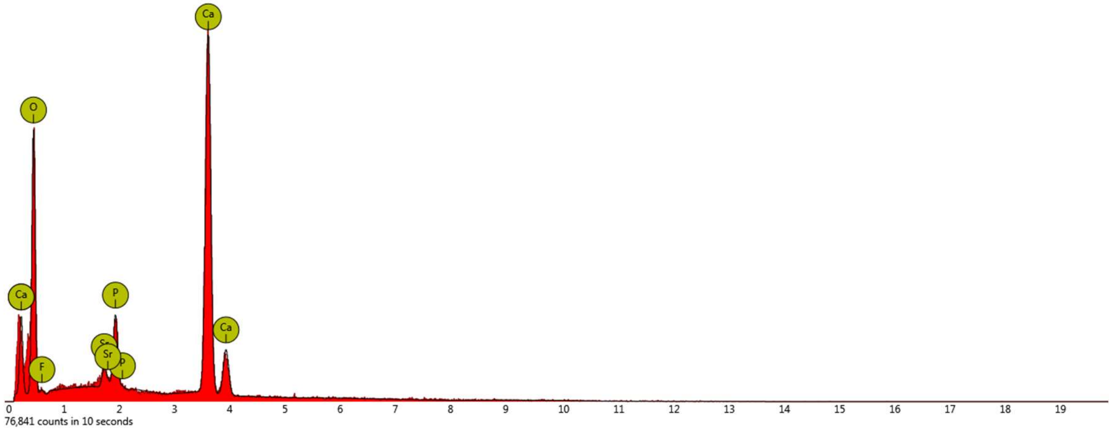

Disabled elements: Am, B, Br, C, Cs, Ho, In, Ir, N, Np, Os, Pm, Pu, Re, Sb, Sn, Tb, Tc, Te, Tm, W

3. spot

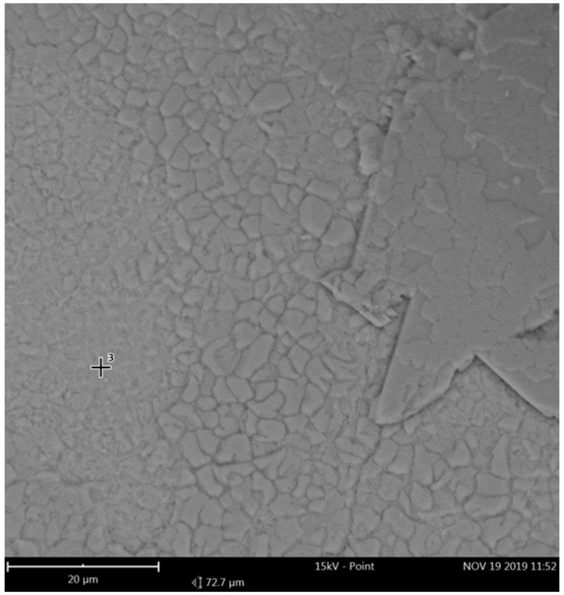

Element Symbol	Atomic Conc.	Weight Conc.	Oxide Symbol	Stoich. wt Conc.
O	67.82	50.52		
Ca	17.45	32.57	CaO	60.38
F	7.76	6.86	F	9.09
P	6.97	10.05	P_2O_5	30.53

FOV: 72.7 µm, Mode: 15kV - Point, Detector: BSD Full, Time: NOV 19 2019 11:52

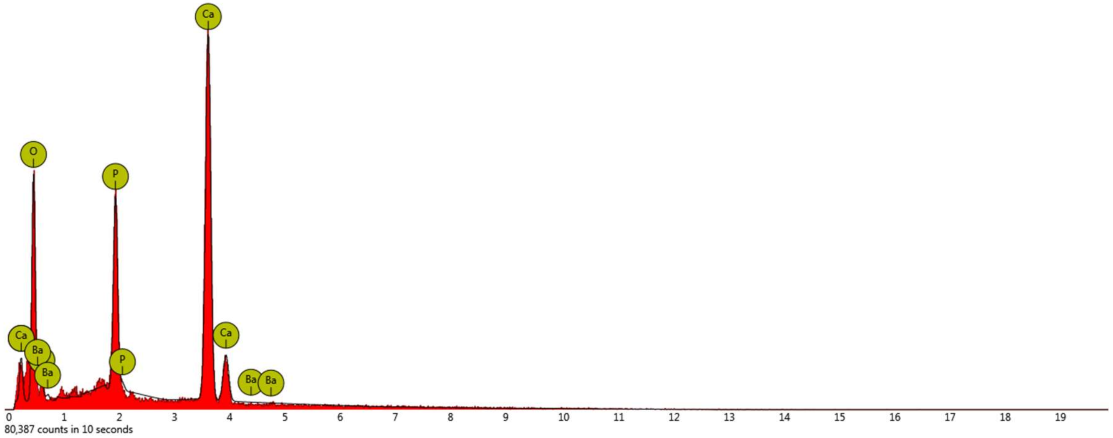

Disabled elements: Am, B, Br, C, Cs, Ho, In, Ir, N, Np, Os, Pm, Pu, Re, Sb, Sn, Tb, Tc, Te, Tm, W

4. spot

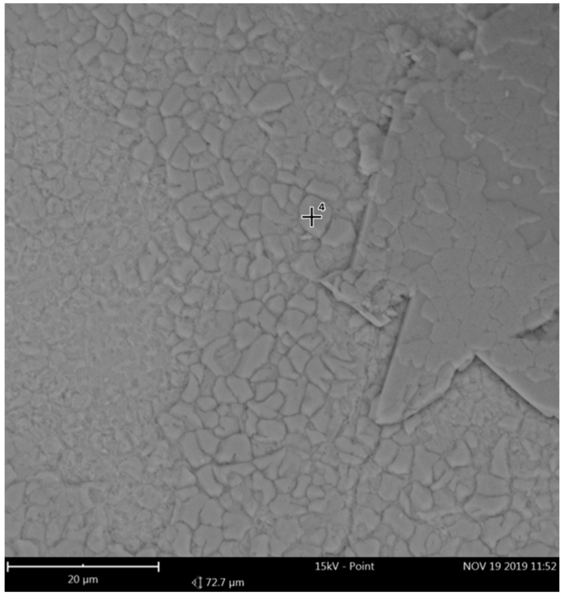

Element Symbol	Atomic Conc.	Weight Conc.	Oxide Symbol	Stoich. wt Conc.
O	80.29	62.78		
Ca	15.62	30.60	CaO	78.33
P	2.51	3.80	P_2O_5	15.92
F	1.18	1.09	F	2.00

FOV: 72.7 µm, Mode: 15kV - Point, Detector: BSD Full, Time: NOV 19 2019 11:52

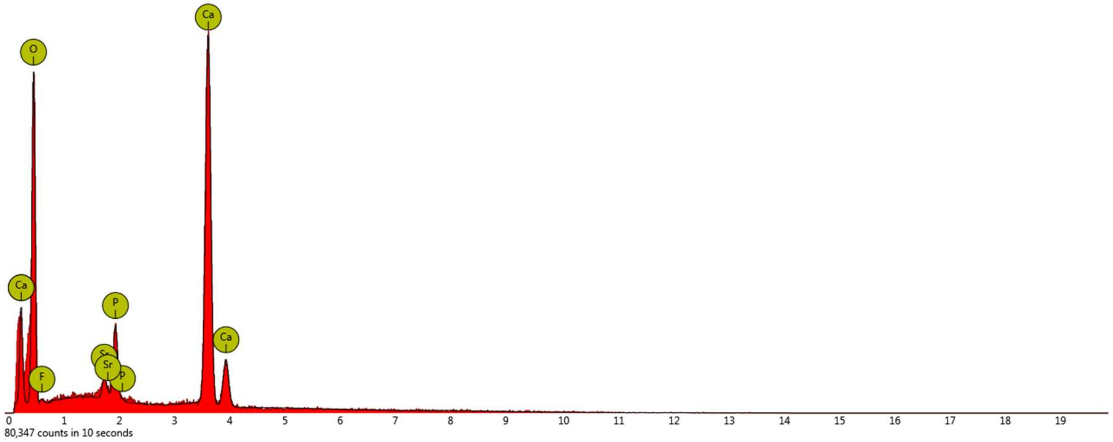

Disabled elements: Am, B, Br, C, Cs, Ho, In, Ir, N, Np, Os, Pm, Pu, Re, Sb, Sn, Tb, Tc, Te, Tm, W

5. spot

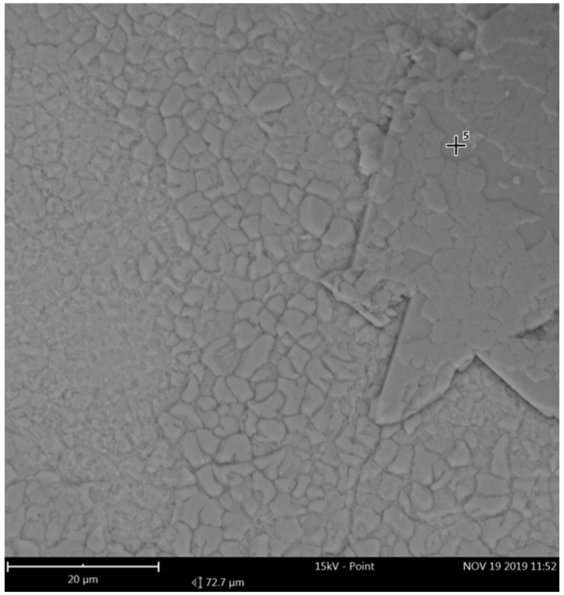

Element Symbol	Atomic Conc.	Weight Conc.	Oxide Symbol	Stoich. wt Conc.
O	80.40	63.58		
Ca	16.43	32.54	CaO	86.81
F	1.64	1.54	F	2.93
P	1.53	2.35	P_2O_5	10.26

FOV: 72.7 µm, Mode: 15kV - Point, Detector: BSD Full, Time: NOV 19 2019 11:52

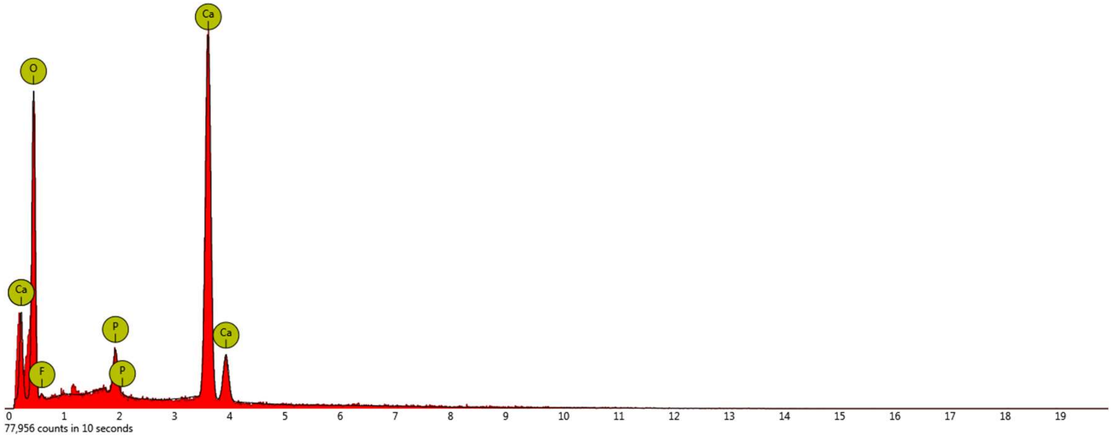

Disabled elements: Am, B, Br, C, Cs, Ho, In, Ir, N, Np, Os, Pm, Pu, Re, Sb, Sn, Tb, Tc, Te, Tm, W

Image 2

1. spot

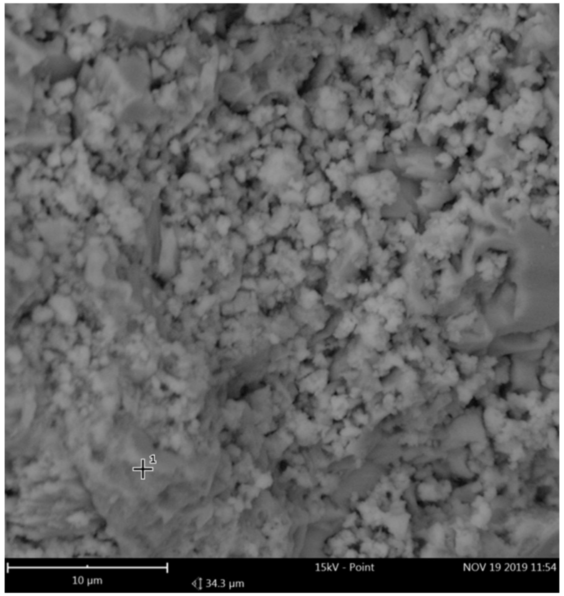

Element Symbol	Atomic Conc.	Weight Conc.	Oxide Symbol	Stoich. wt Conc.
O	55.68	33.88		
Ca	33.90	51.67	CaO	75.59
Fe	3.75	7.96	Fe_2O_3	11.90
P	3.04	3.58	P_2O_5	8.57
F	2.22	1.61	F	1.68
Mg	1.41	1.30	MgO	2.25

FOV: 34.3 µm, Mode: 15kV - Point, Detector: BSD Full, Time: NOV 19 2019 11:54

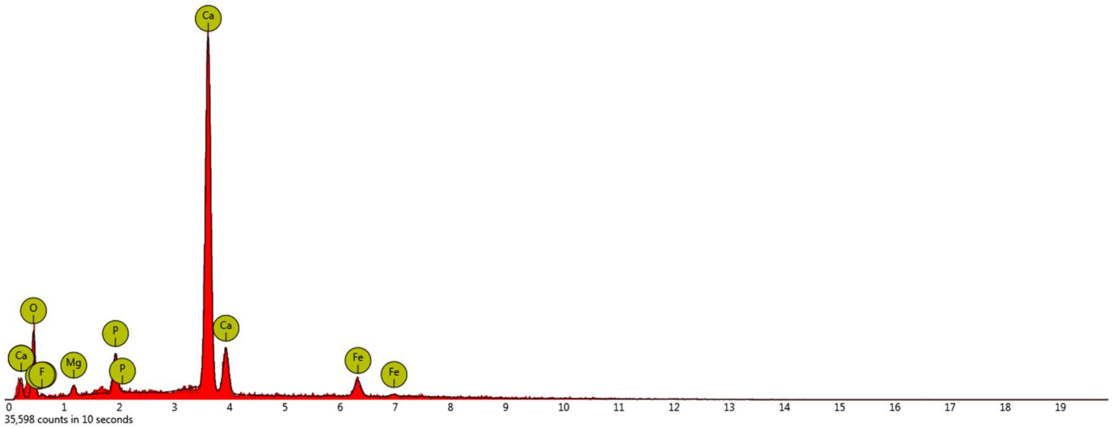

Disabled elements: Am, B, Br, C, Cs, Ho, In, Ir, N, Np, Os, Pm, Pu, Re, Sb, Sn, Tb, Tc, Te, Tm, W

2. spot

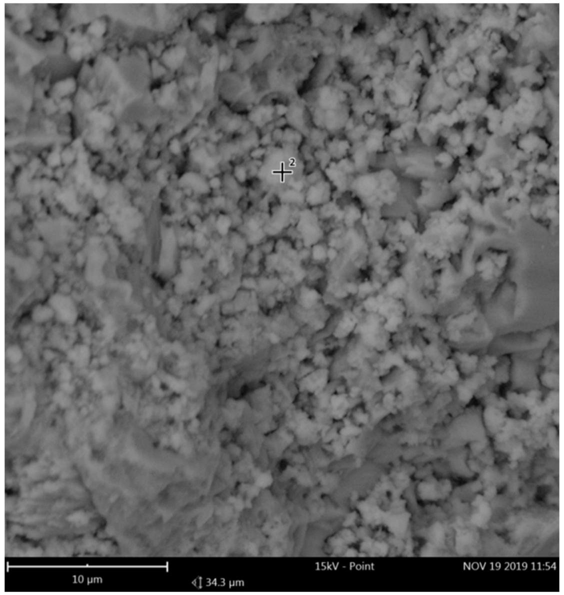

Element Symbol	Atomic Conc.	Weight Conc.	Oxide Symbol	Stoich. wt Conc.
O	56.80	35.74		
Ca	34.28	54.04	CaO	81.17
P	4.66	5.68	P_2O_5	13.96
F	3.94	2.95	F	3.16

FOV: 34.3 µm, Mode: 15kV - Point, Detector: BSD Full, Time: NOV 19 2019 11:54

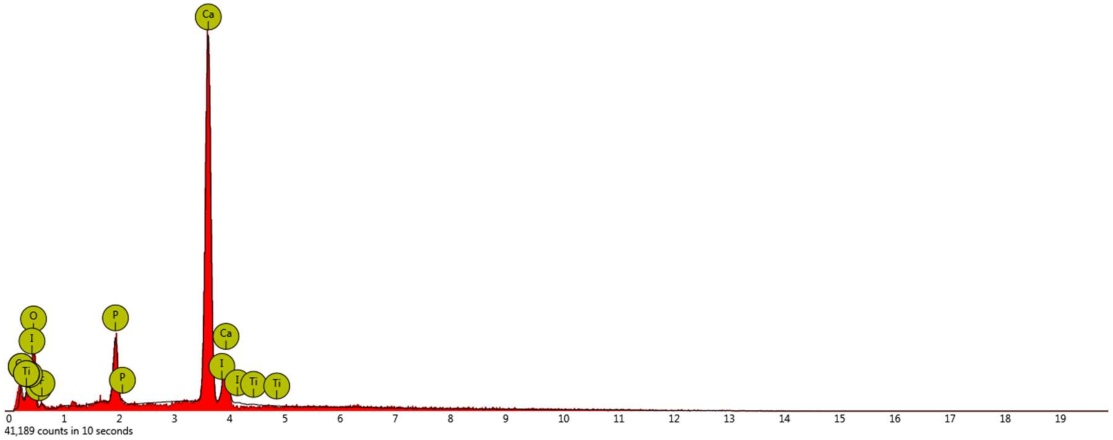

Disabled elements: Am, B, Br, C, Cs, Ho, In, Ir, N, Np, Os, Pm, Pu, Re, Sb, Sn, Tb, Tc, Te, Tm, W

Supplementary Data S2. SEM-EDS report for sample NHMW 2020/0033/0002 1910A.

1910A

Contains 8 images with a total of 17 analyses

01. Image 1
3 analyses: 3x spot

02. Image 2
1 analysis: 1x spot

03. Image 3
1 analysis: 1x spot

04. Image 4
1 analysis: 1x spot

05. Image 5
8 analyses: 8x spot

06. Image 6
1 analysis: 1x spot

07. Image 7
1 analysis: 1x spot

08. Image 8

1 analysis: 1x spot

Image 1

1. spot

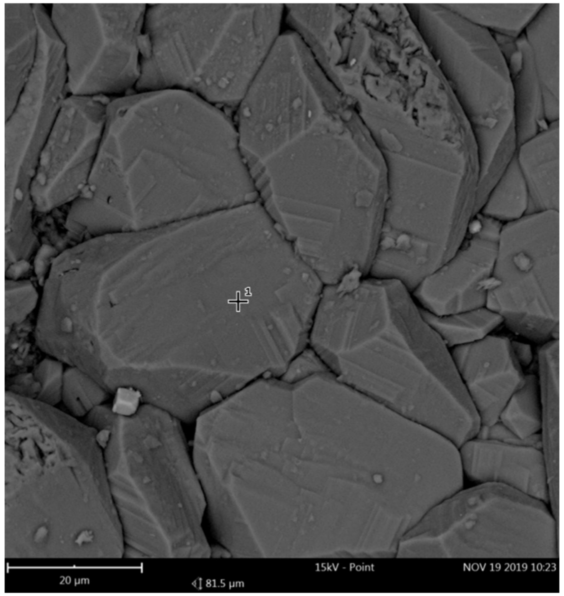

Element Symbol	Atomic Conc.	Weight Conc.	Oxide Symbol	Stoich. wt Conc.
O	65.08	43.12		
Ca	33.00	54.77	CaO	94.94
Si	0.80	0.93	SiO_2	2.46
Mg	0.59	0.59	MgO	1.22
Al	0.53	0.59	Al_2O_3	1.39

FOV: 81.5 µm, Mode: 15kV - Point, Detector: BSD Full, Time: NOV 19 2019 10:23

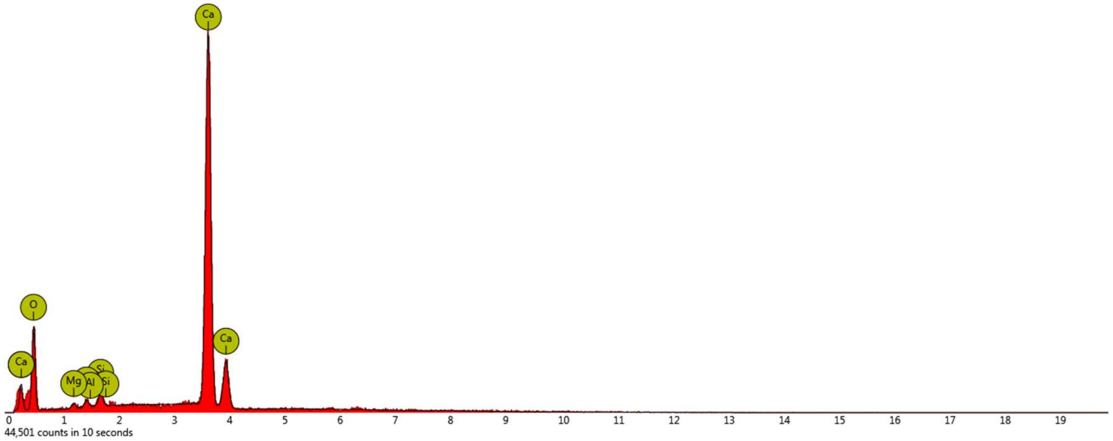

Disabled elements: Am, B, Br, C, Cs, Ho, In, Ir, N, Np, Os, Pm, Pu, Re, Sb, Sn, Tb, Tc, Te, Tm, W

2. spot

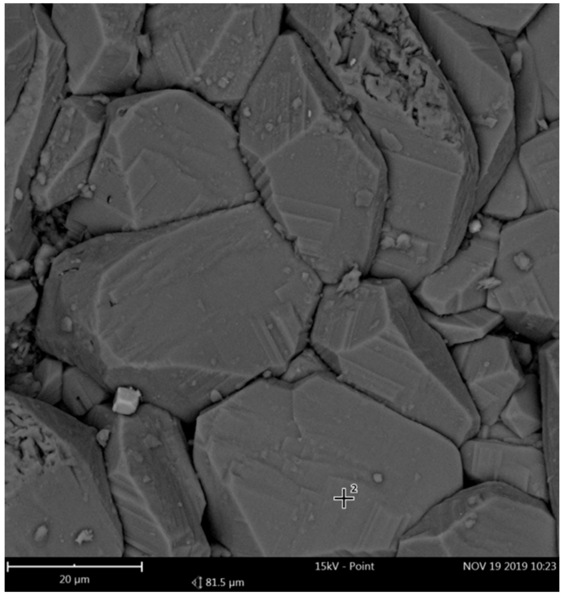

Element Symbol	Atomic Conc.	Weight Conc.	Oxide Symbol	Stoich. wt Conc.
O	69.58	48.31		
Ca	28.35	49.31	CaO	93.77
Si	0.82	1.00	SiO_2	2.90
Mg	0.66	0.70	MgO	1.58
Al	0.58	0.68	Al_2O_3	1.75

FOV: 81.5 µm, Mode: 15kV - Point, Detector: BSD Full, Time: NOV 19 2019 10:23

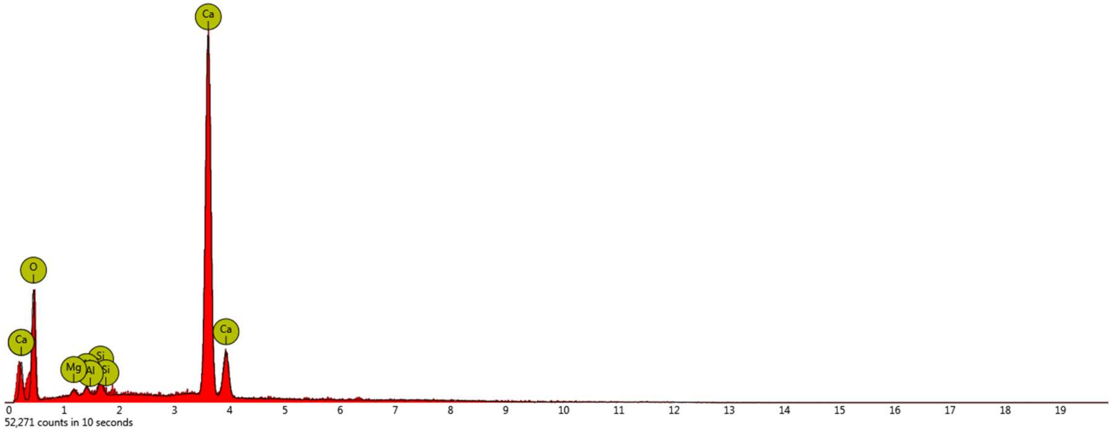

Disabled elements: Am, B, Br, C, Cs, Ho, In, Ir, N, Np, Os, Pm, Pu, Re, Sb, Sn, Tb, Tc, Te, Tm, W

3. spot

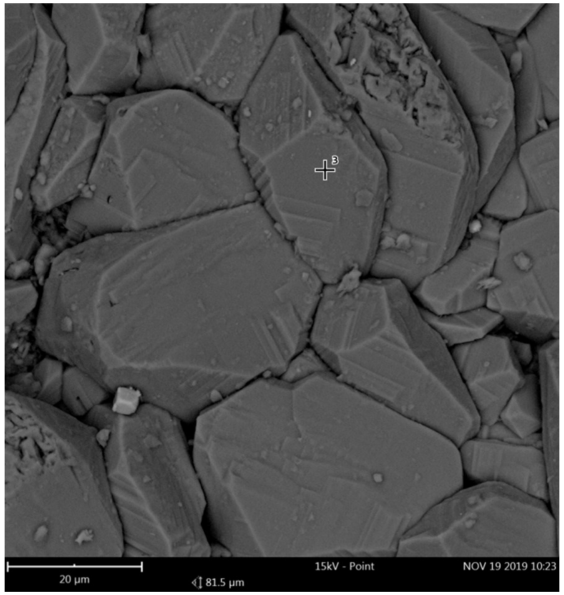

Element Symbol	Atomic Conc.	Weight Conc.	Oxide Symbol	Stoich. wt Conc.
O	74.78	54.79		
Ca	23.50	43.13	CaO	93.77
Si	0.71	0.91	SiO_2	3.04
Mg	0.61	0.68	MgO	1.76
Al	0.39	0.49	Al_2O_3	1.43

FOV: 81.5 µm, Mode: 15kV - Point, Detector: BSD Full, Time: NOV 19 2019 10:23

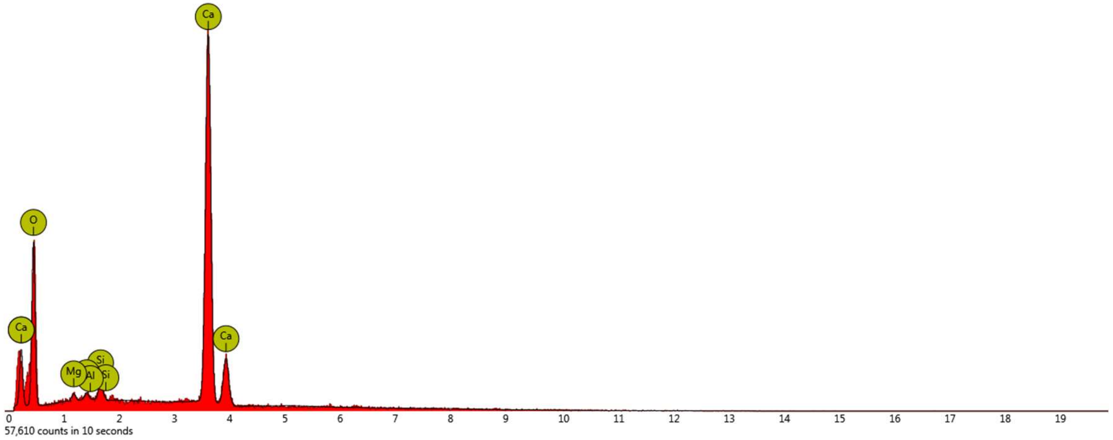

Disabled elements: Am, B, Br, C, Cs, Ho, In, Ir, N, Np, Os, Pm, Pu, Re, Sb, Sn, Tb, Tc, Te, Tm, W

Image 2

1. spot

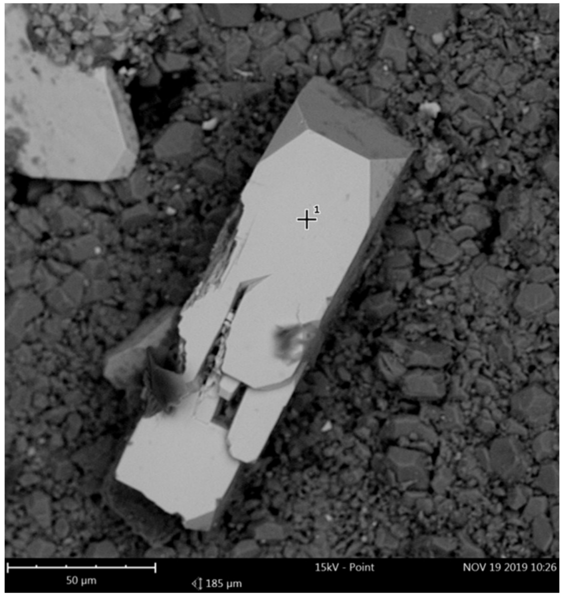

Element Symbol	Atomic Conc.	Weight Conc.	Oxide Symbol	Stoich. wt Conc.
O	78.64	42.99		
S	9.34	10.23	SO_3	32.38
Ba	9.13	42.82	BaO	60.59
Ca	2.89	3.96	CaO	7.03

FOV: 185 µm, Mode: 15kV - Point, Detector: BSD Full, Time: NOV 19 2019 10:26

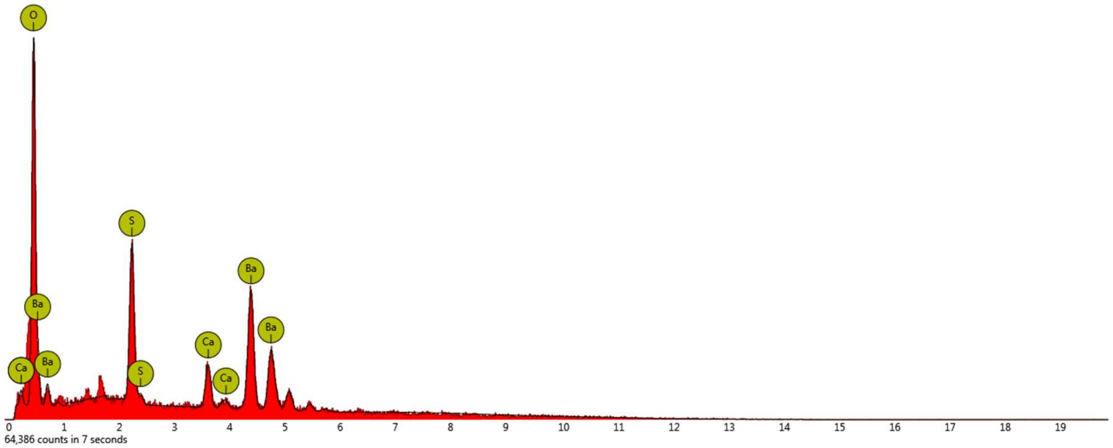

Disabled elements: Am, B, Br, C, Cs, Ho, In, Ir, N, Np, Os, Pm, Pu, Re, Sb, Sn, Tb, Tc, Te, Tm, W

Image 3

1. spot

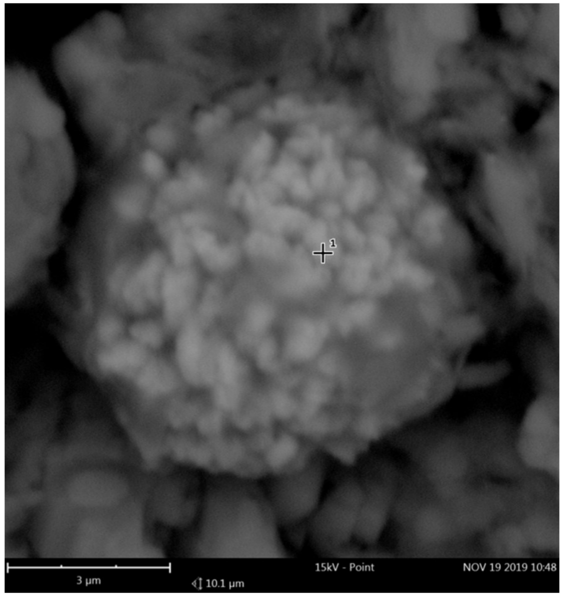

Element Symbol	Atomic Conc.	Weight Conc.	Oxide Symbol	Stoich. wt Conc.
O	58.10	36.45		
S	22.01	27.68	SO_3	55.94
Fe	11.00	24.09	Fe_2O_3	27.87
Ca	3.92	6.17	CaO	6.98
Si	3.18	3.50	SiO_2	6.06
Al	1.50	1.59	Al_2O_3	2.43

FOV: 10.1 µm, Mode: 15kV - Point, Detector: BSD Full, Time: NOV 19 2019 10:48

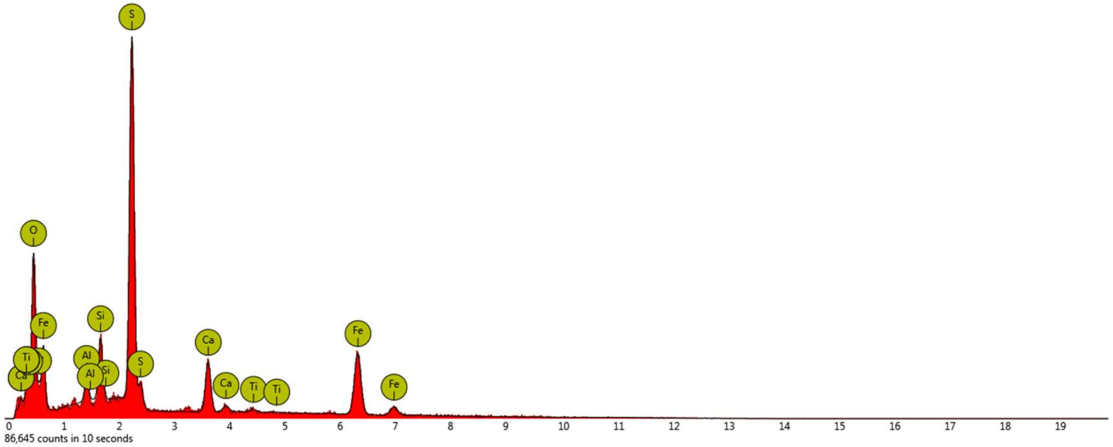

Disabled elements: Am, B, Br, C, Cs, Ho, In, Ir, N, Np, Os, Pm, Pu, Re, Sb, Sn, Tb, Tc, Te, Tm, W

Image 4

1. spot

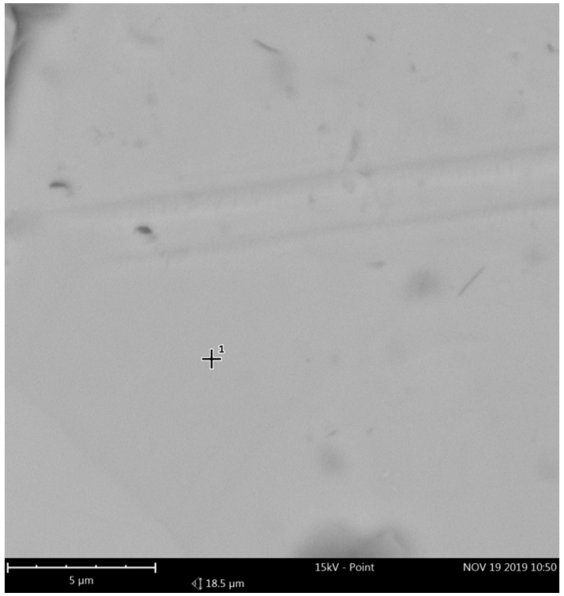

Element Symbol	Atomic Conc.	Weight Conc.	Oxide Symbol	Stoich. wt Conc.
O	75.91	41.22		
Ba	9.07	42.29	BaO	56.79
S	8.87	9.65	SO_3	28.99
Ca	2.50	3.41	CaO	5.73
Si	2.41	2.30	SiO_2	5.92
Al	1.23	1.13	Al_2O_3	2.57

FOV: 18.5 µm, Mode: 15kV - Point, Detector: BSD Full, Time: NOV 19 2019 10:50

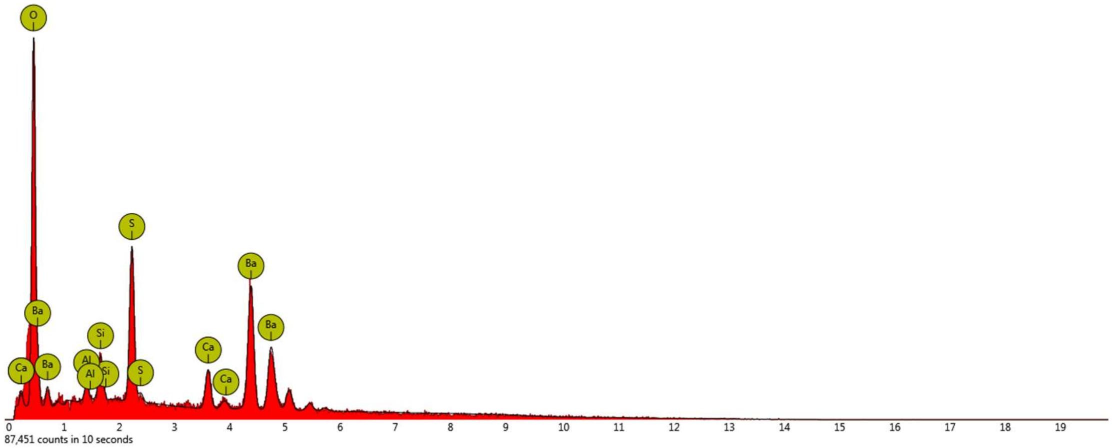

Disabled elements: Am, B, Br, C, Cs, Ho, In, Ir, N, Np, Os, Pm, Pu, Re, Sb, Sn, Tb, Tc, Te, Tm, W

Image 5

1. spot

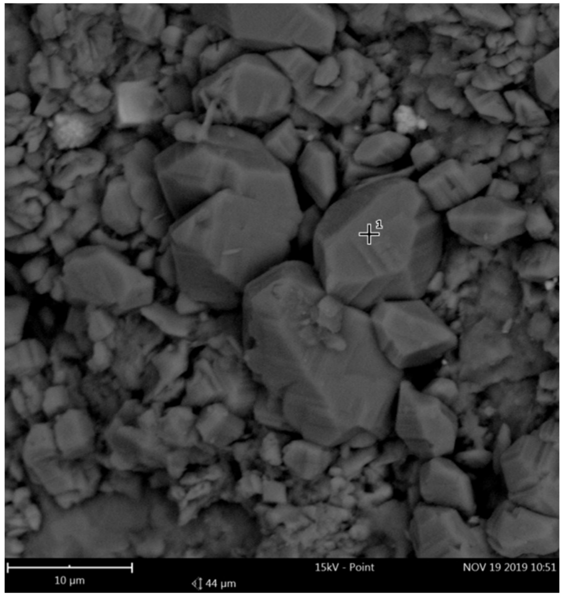

Element Symbol	Atomic Conc.	Weight Conc.	Oxide Symbol	Stoich. wt Conc.
O	75.83	57.26		
Ca	18.42	34.84	CaO	76.21
Si	2.47	3.27	SiO_2	10.94
Al	1.41	1.80	Al_2O_3	5.31
Mg	0.86	0.98	MgO	2.55
K	0.41	0.77	K_2O	1.44
S	0.38	0.57	SO_3	2.24

FOV: 44 µm, Mode: 15kV - Point, Detector: BSD Full, Time: NOV 19 2019 10:51

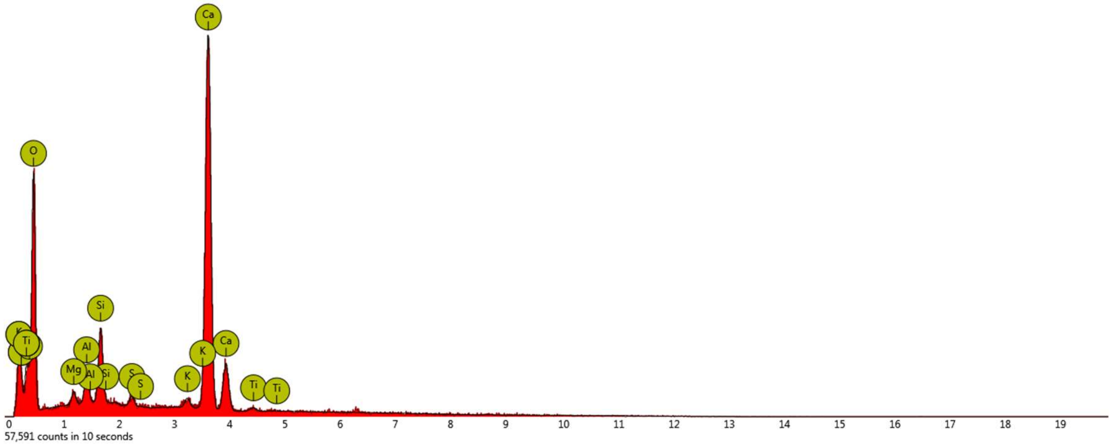

Disabled elements: Am, B, Br, C, Cs, Ho, In, Ir, N, Np, Os, Pm, Pu, Re, Sb, Sn, Tb, Tc, Te, Tm, W

2. spot

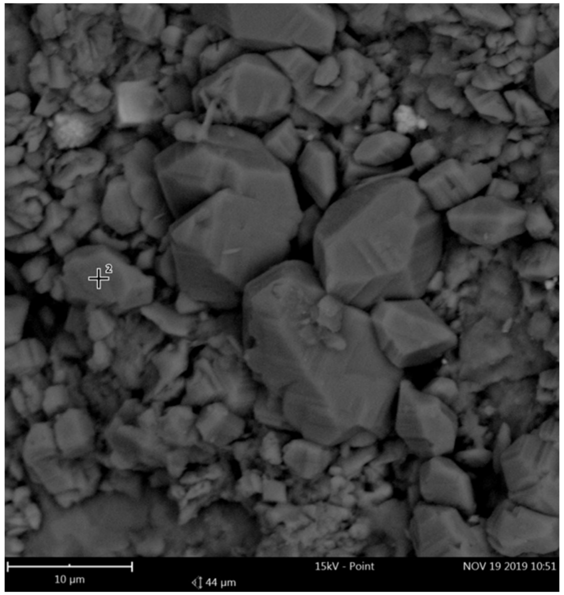

Element Symbol	Atomic Conc.	Weight Conc.	Oxide Symbol	Stoich. wt Conc.
O	80.75	63.97		
Ca	14.22	28.22	CaO	73.73
Si	1.97	2.73	SiO_2	10.92
Al	1.29	1.72	Al_2O_3	6.06
Mg	1.06	1.28	MgO	3.96
K	0.35	0.68	K_2O	1.54
S	0.21	0.34	SO_3	1.57

FOV: 44 μm, Mode: 15kV - Point, Detector: BSD Full, Time: NOV 19 2019 10:51

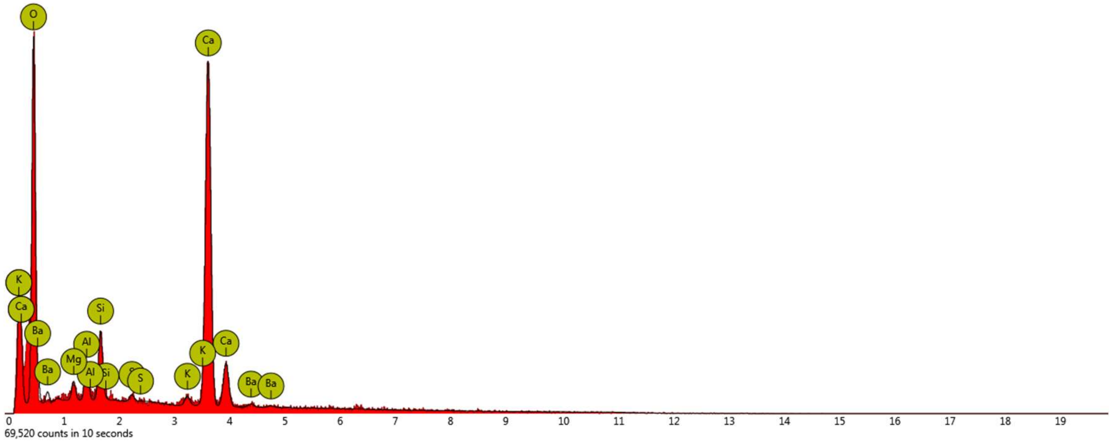

Disabled elements: Am, B, Br, C, Cs, Ho, In, Ir, N, Np, Os, Pm, Pu, Re, Sb, Sn, Tb, Tc, Te, Tm, W

3. spot

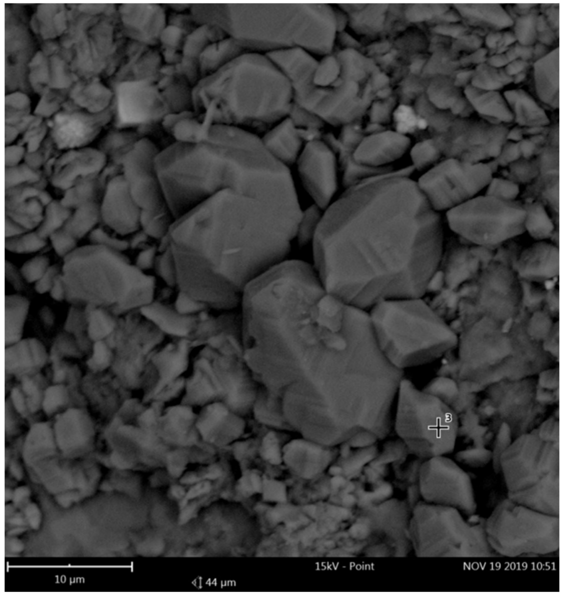

Element Symbol	Atomic Conc.	Weight Conc.	Oxide Symbol	Stoich. wt Conc.
O	77.86	60.16		
Ca	16.49	31.92	CaO	74.37
Si	2.53	3.43	SiO_2	12.22
Al	1.35	1.76	Al_2O_3	5.54
Mg	0.86	1.01	MgO	2.78
S	0.36	0.55	SO_3	2.30
K	0.30	0.57	K_2O	1.14

FOV: 44 µm, Mode: 15kV - Point, Detector: BSD Full, Time: NOV 19 2019 10:51

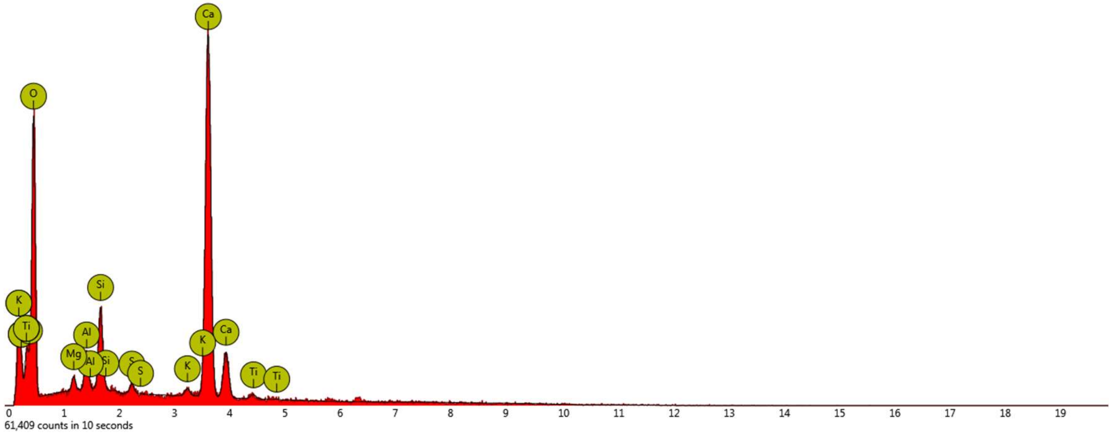

Disabled elements: Am, B, Br, C, Cs, Ho, In, Ir, N, Np, Os, Pm, Pu, Re, Sb, Sn, Tb, Tc, Te, Tm, W

4. spot

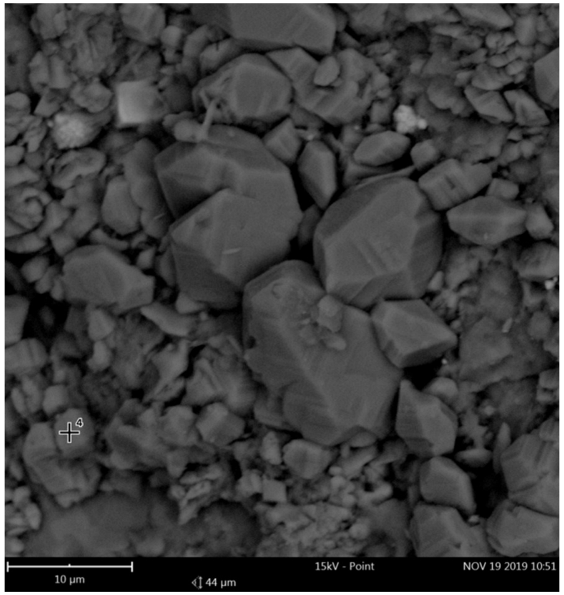

Element Symbol	Atomic Conc.	Weight Conc.	Oxide Symbol	Stoich. wt Conc.
O	80.92	64.67		
Ca	14.38	28.79	CaO	75.99
Si	2.00	2.81	SiO_2	11.33
Al	1.24	1.67	Al_2O_3	5.95
Mg	0.94	1.14	MgO	3.58
S	0.27	0.43	SO_3	2.02
K	0.25	0.49	K_2O	1.12

FOV: 44 µm, Mode: 15kV - Point, Detector: BSD Full, Time: NOV 19 2019 10:51

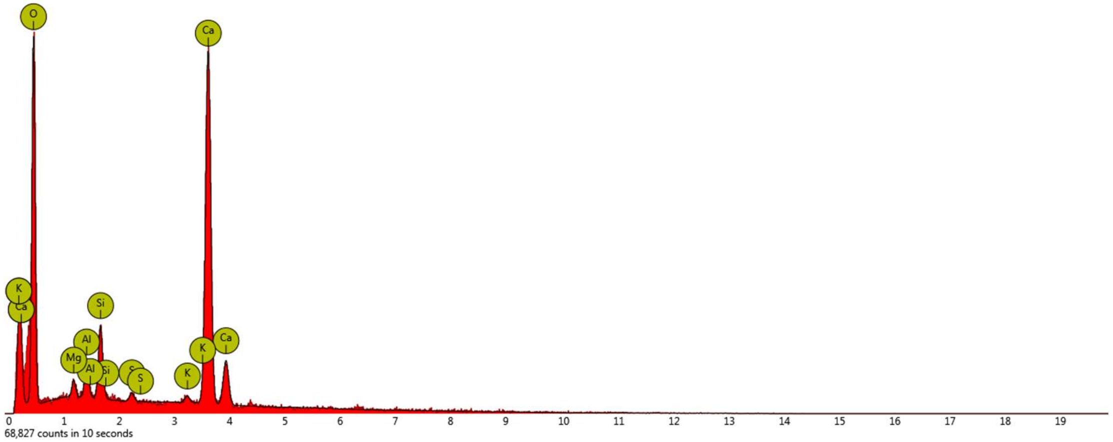

Disabled elements: Am, B, Br, C, Cs, Ho, In, Ir, N, Np, Os, Pm, Pu, Re, Sb, Sn, Tb, Tc, Te, Tm, W

5. spot

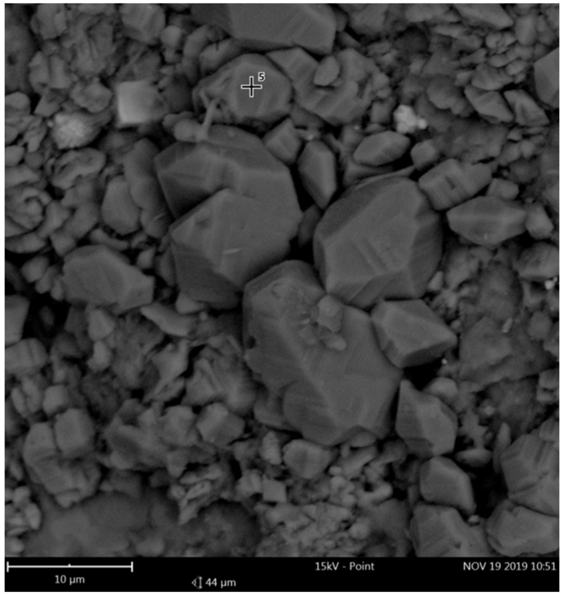

Element Symbol	Atomic Conc.	Weight Conc.	Oxide Symbol	Stoich. wt Conc.
O	75.50	56.92		
Ca	18.77	35.45	CaO	76.96
Si	2.56	3.38	SiO_2	11.23
Al	1.60	2.03	Al_2O_3	5.95
Mg	0.95	1.09	MgO	2.79
K	0.27	0.50	K_2O	0.93
S	0.25	0.37	SO_3	1.45

FOV: 44 µm, Mode: 15kV - Point, Detector: BSD Full, Time: NOV 19 2019 10:51

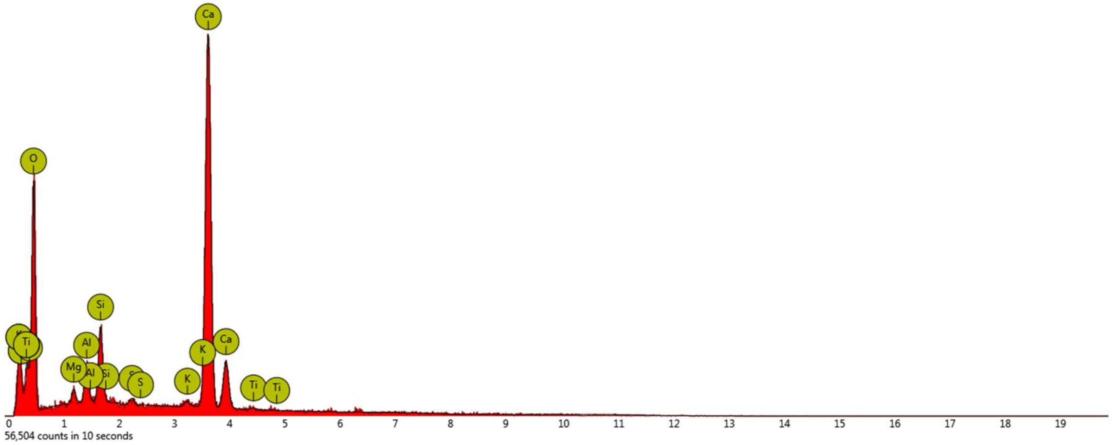

Disabled elements: Am, B, Br, C, Cs, Ho, In, Ir, N, Np, Os, Pm, Pu, Re, Sb, Sn, Tb, Tc, Te, Tm, W

6. spot

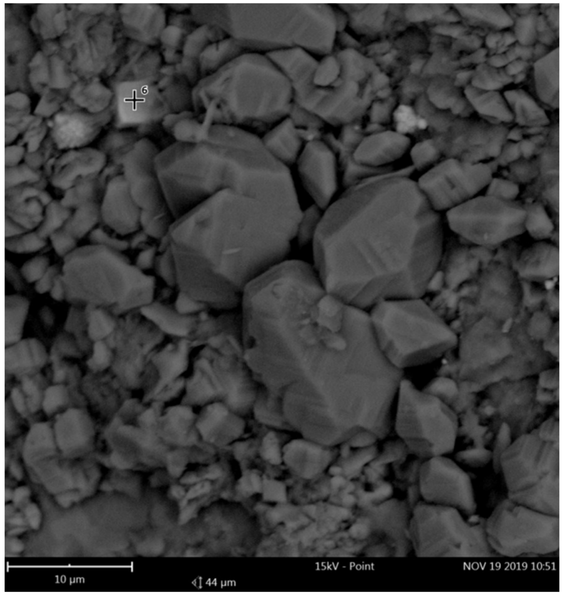

Element Symbol	Atomic Conc.	Weight Conc.	Oxide Symbol	Stoich. wt Conc.
O	64.28	44.08		
S	13.62	18.72	SO_3	44.05
Fe	7.47	17.88	Fe_2O_3	24.10
Si	4.82	5.80	SiO_2	11.69
Ca	4.31	7.40	CaO	9.76
Al	3.33	3.85	Al_2O_3	6.86
Mg	2.18	2.27	MgO	3.54

FOV: 44 µm, Mode: 15kV - Point, Detector: BSD Full, Time: NOV 19 2019 10:51

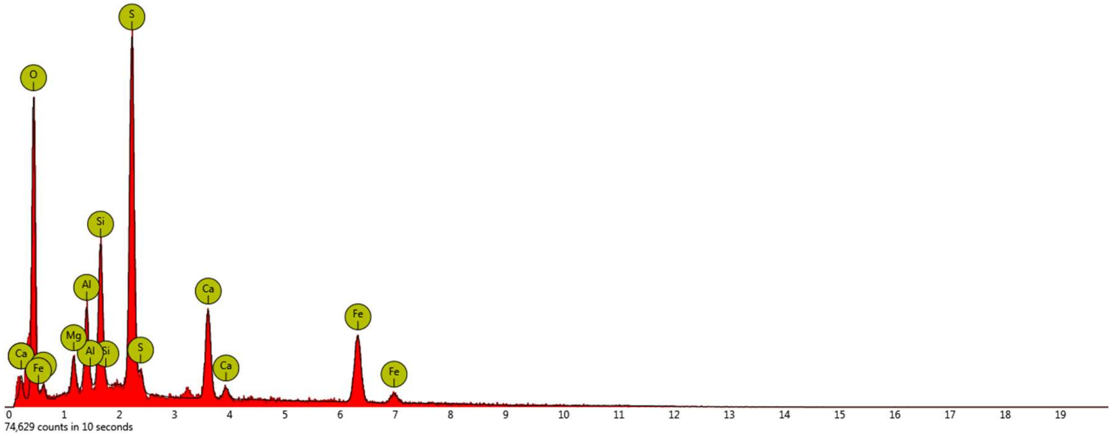

Disabled elements: Am, B, Br, C, Cs, Ho, In, Ir, N, Np, Os, Pm, Pu, Re, Sb, Sn, Tb, Tc, Te, Tm, W

7. spot

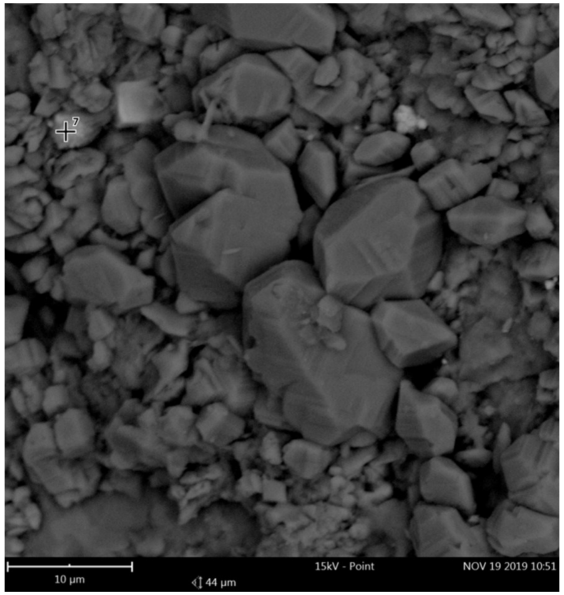

Element Symbol	Atomic Conc.	Weight Conc.	Oxide Symbol	Stoich. wt Conc.
O	54.15	32.96		
S	25.14	30.67	SO_3	58.35
Fe	11.44	24.32	Fe_2O_3	26.49
Ca	4.86	7.41	CaO	7.90
Si	2.81	3.00	SiO_2	4.89
Al	1.60	1.64	Al_2O_3	2.37

FOV: 44 µm, Mode: 15kV - Point, Detector: BSD Full, Time: NOV 19 2019 10:51

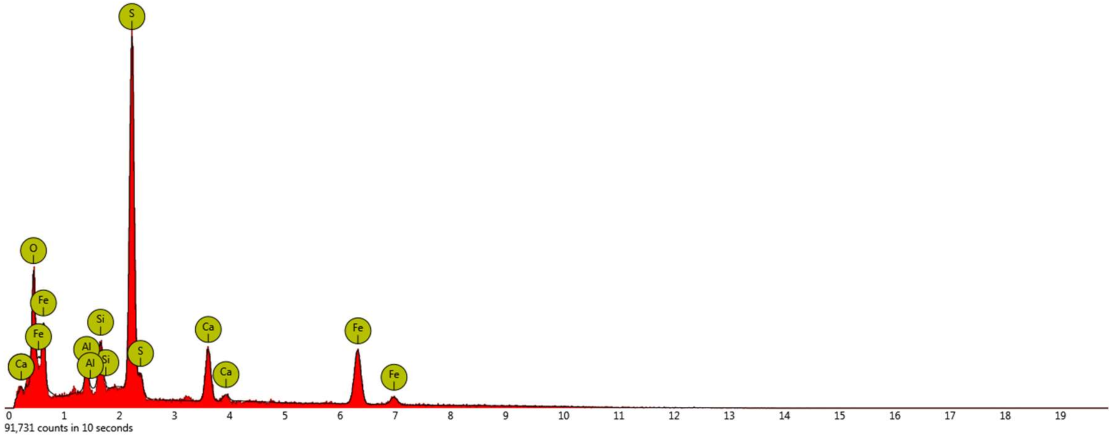

Disabled elements: Am, B, Br, C, Cs, Ho, In, Ir, N, Np, Os, Pm, Pu, Re, Sb, Sn, Tb, Tc, Te, Tm, W

8. spot

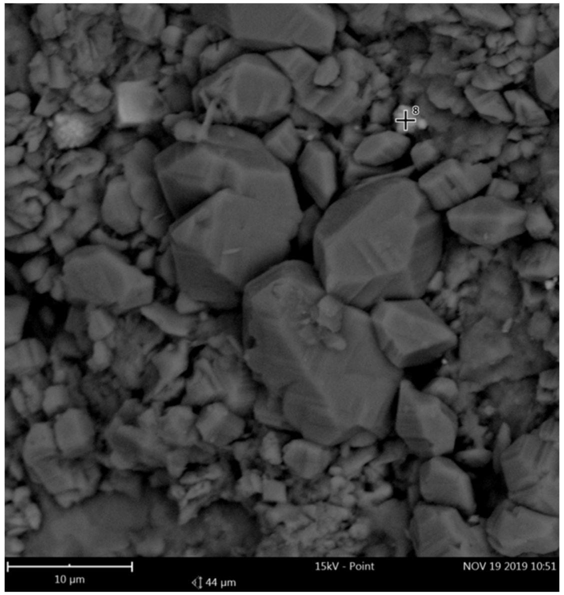

Element Symbol	Atomic Conc.	Weight Conc.	Oxide Symbol	Stoich. wt Conc.
O	51.74	30.89		
S	27.14	32.48	SO_3	59.68
Fe	11.87	24.73	Fe_2O_3	26.02
Ca	5.08	7.59	CaO	7.82
Si	2.62	2.74	SiO_2	4.32
Al	1.55	1.56	Al_2O_3	2.17

FOV: 44 µm, Mode: 15kV - Point, Detector: BSD Full, Time: NOV 19 2019 10:51

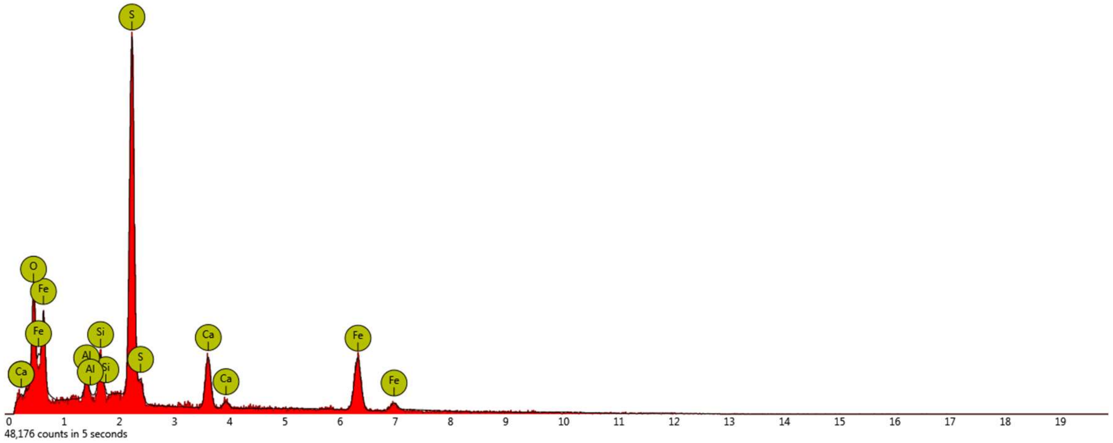

Disabled elements: Am, B, Br, C, Cs, Ho, In, Ir, N, Np, Os, Pm, Pu, Re, Sb, Sn, Tb, Tc, Te, Tm, W

Image 6

1. spot

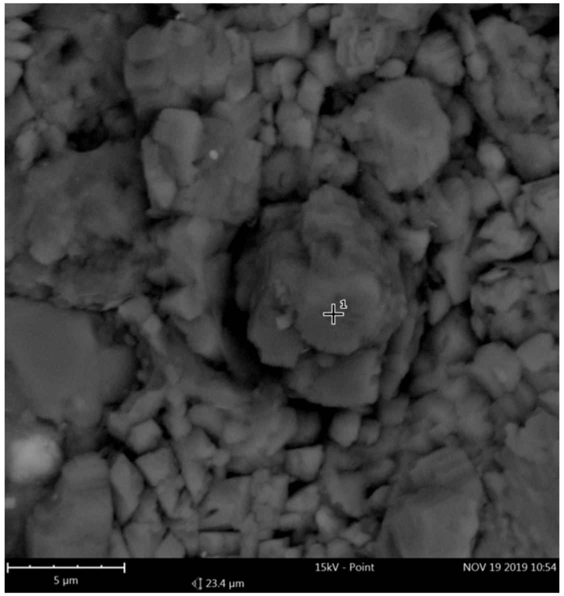

Element Symbol	Atomic Conc.	Weight Conc.	Oxide Symbol	Stoich. wt Conc.
O	77.27	63.21		
Ca	9.79	20.05	CaO	48.44
Mg	9.15	11.37	MgO	32.55
Si	2.41	3.46	SiO_2	12.79
Al	1.38	1.91	Al_2O_3	6.22

FOV: 23.4 µm, Mode: 15kV - Point, Detector: BSD Full, Time: NOV 19 2019 10:54

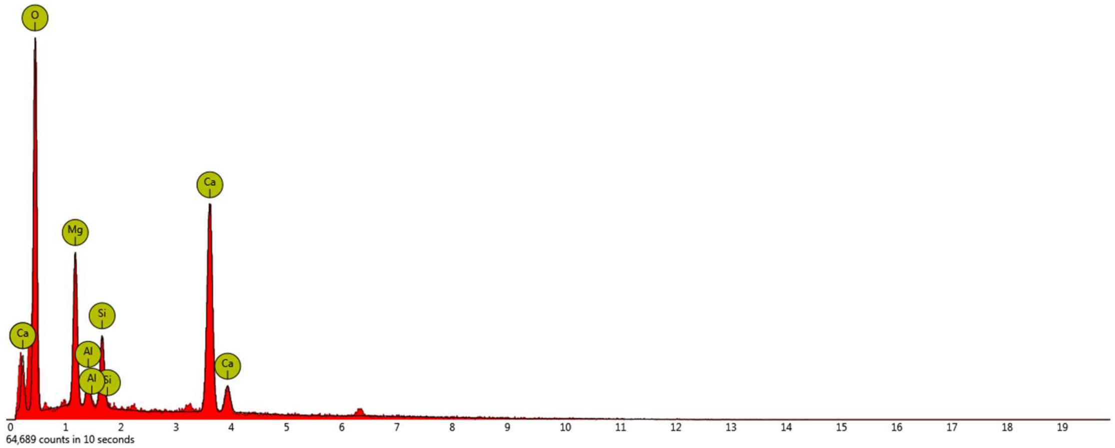

Disabled elements: Am, B, Br, C, Cs, Ho, In, Ir, N, Np, Os, Pm, Pu, Re, Sb, Sn, Tb, Tc, Te, Tm, W

Image 7

1. spot

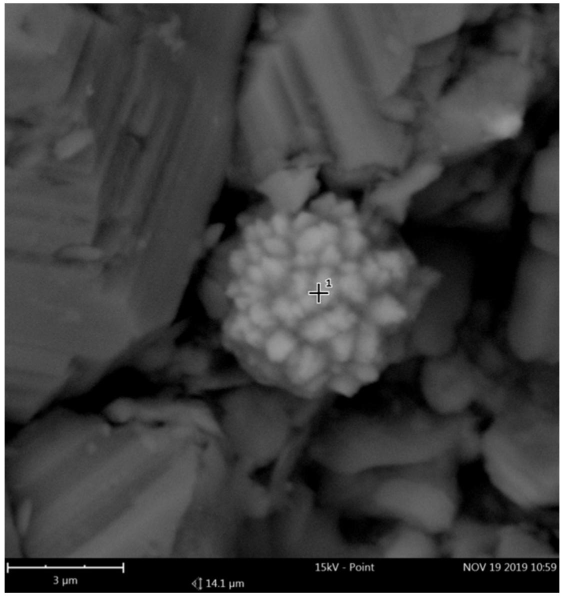

Element Symbol	Atomic Conc.	Weight Conc.	Oxide Symbol	Stoich. wt Conc.
O	54.87	30.09		
Fe	20.01	38.31	Fe_2O_3	50.91
Ca	16.38	22.49	CaO	29.26
S	5.50	6.04	SO_3	14.03
Si	1.88	1.81	SiO_2	3.60
Al	1.36	1.26	Al_2O_3	2.21

FOV: 14.1 µm, Mode: 15kV - Point, Detector: BSD Full, Time: NOV 19 2019 10:59

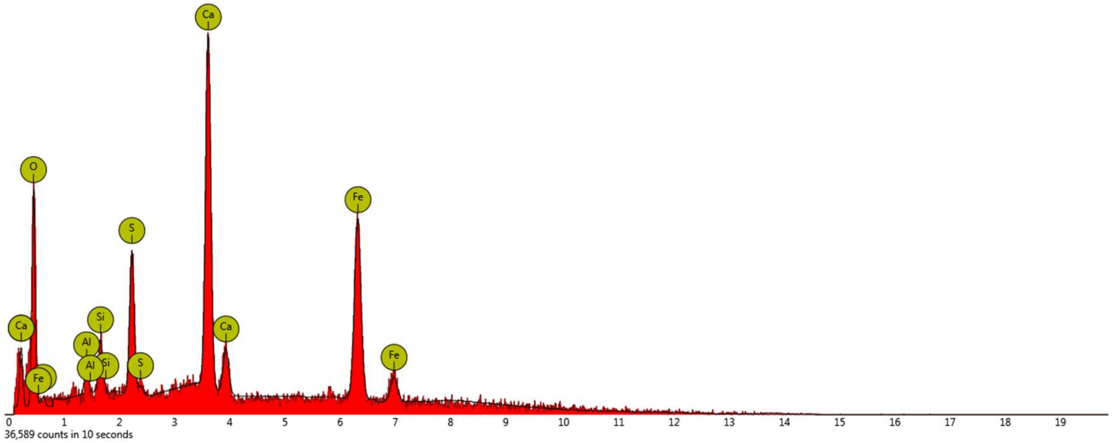

Disabled elements: Am, B, Br, C, Cs, Ho, In, Ir, N, Np, Os, Pm, Pu, Re, Sb, Sn, Tb, Tc, Te, Tm, W

Image 8

1. spot

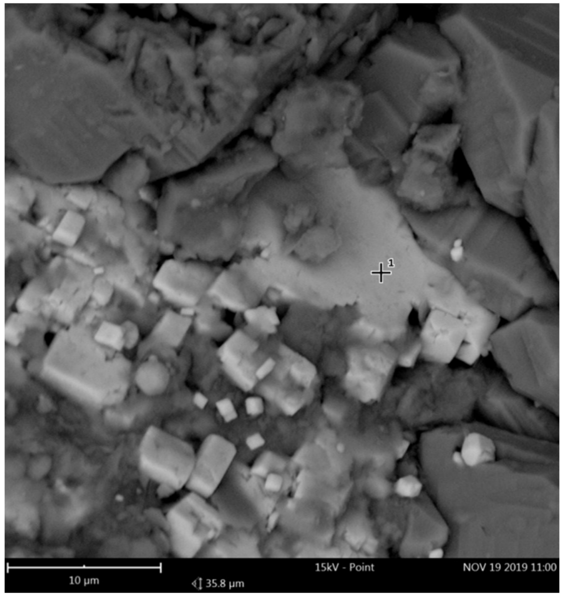

Element Symbol	Atomic Conc.	Weight Conc.	Oxide Symbol	Stoich. wt Conc.
O	71.48	42.98		
Ca	18.08	27.23	CaO	49.00
Ba	4.55	23.50	BaO	33.73
Si	3.16	3.33	SiO_2	9.17
Al	1.73	1.76	Al_2O_3	4.26
S	0.99	1.19	SO_3	3.83

FOV: 35.8 µm, Mode: 15kV - Point, Detector: BSD Full, Time: NOV 19 2019 11:00

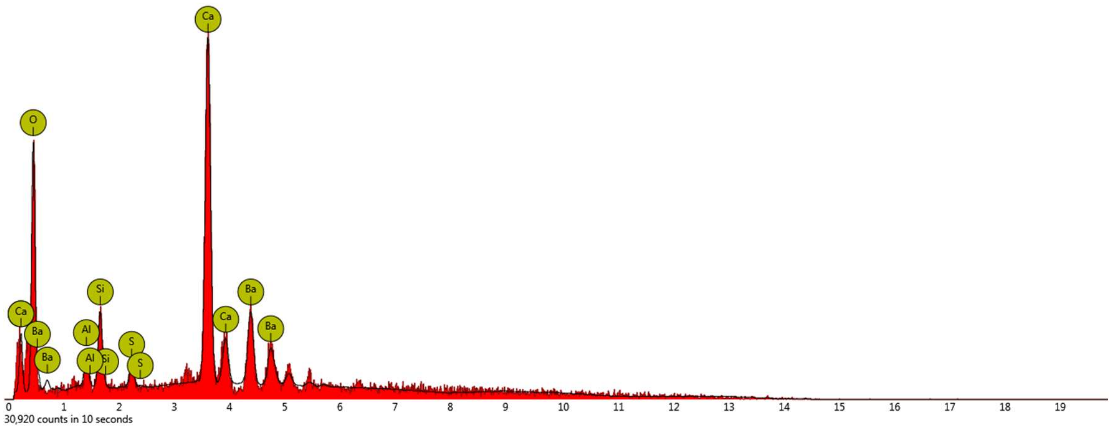

Disabled elements: Am, B, Br, C, Cs, Ho, In, Ir, N, Np, Os, Pm, Pu, Re, Sb, Sn, Tb, Tc, Te, Tm, W

Supplementary Data S3. XRD report for sample NHMW 2020/0033/0007 AS193.

Host Rock

No.	Ref. Code	Mineral Name	Chemical Formula	Score	SemiQuant [%]
1	01-087-2096	Quartz	Si O2	66	34,5
2	04-007-2613	Pyrite	Fe S2	49	2,5
3	01-089-6426	Albite	Na (Al Si3 O8)	33	13,5
4	04-013-2116	Calcite	Ca0.936 Mg0.064 (C O3)	31	12,5
5	98-002-8098	Clinochlore	H8 Al2.651 Fe1.686 Mg2.96 O18 Si2.622	23	7,5
6	01-087-0691	Muscovite	(K0.94 Na0.06) (Mg0.08 Al1.75 Fe0.15 Mn0.02) (Al0.92 Si3.08) O10 (O H)2	26	8
7	98-005-7001	Illite	H3 Al4 K1 O12 Si2	22	18,5
8	98-001-2020	Dolomite	C2 Ca1 Mg1 O6	14	2,5
9	98-001-7136	Kaolinite	H4 Al2 O9 Si2	11	0,5
					100

Coprolite

No.	Ref. Code	Mineral Name	Chemical Formula	Score	SemiQuant [%]
1	01-073-9696	Carbonate-fluorapatite	Ca9.55 (P O4)4.96 F1.96 (C O3)1.283	70	53
2	01-089-1305	Calcite	(Mg0.06 Ca0.94) (C O3)	59	41,5
3	01-087-2096	Quartz	Si O2	22	2
4	98-002-8098	Clinochlore	H8 Al2.651 Fe1.686 Mg2.96 O18 Si2.622	11	1
5	98-005-7001	Illite	H3 Al4 K1 O12 Si2	10	2,5
					100

verte!

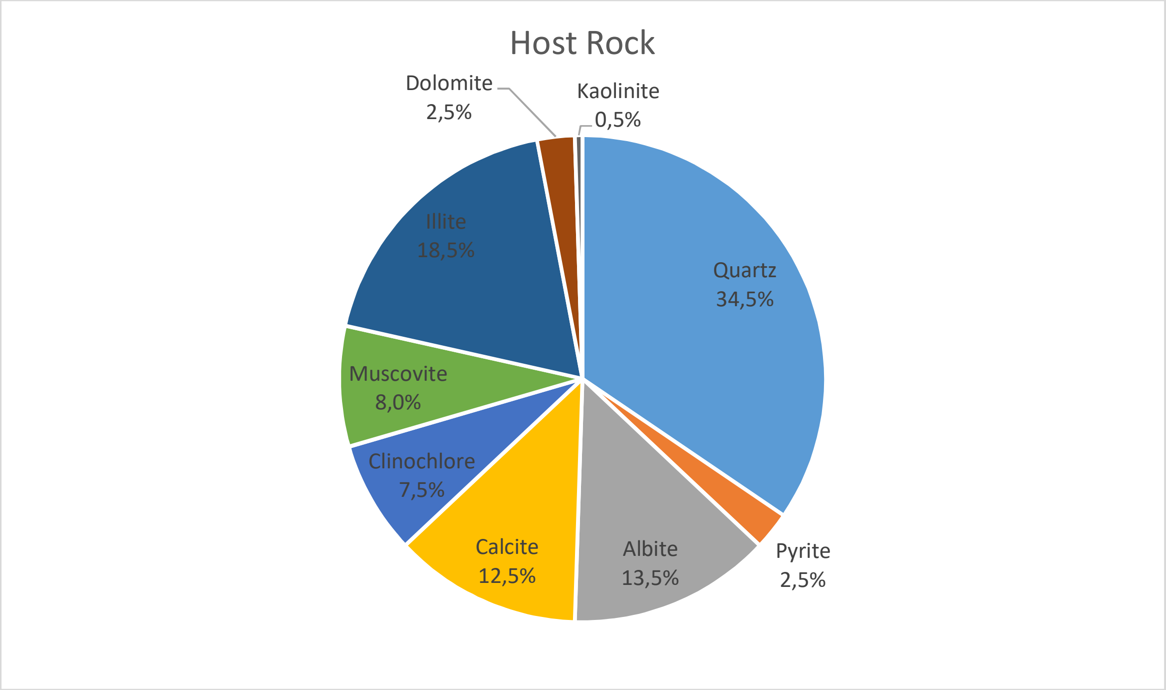
Host Rock
Dolomite 2,5%
Kaolinite 0,5%
Illite 18,5%
Quartz 34,5%
Muscovite 8,0%
Clinochlore 7,5%
Calcite 12,5%
Albite 13,5%
Pyrite 2,5%

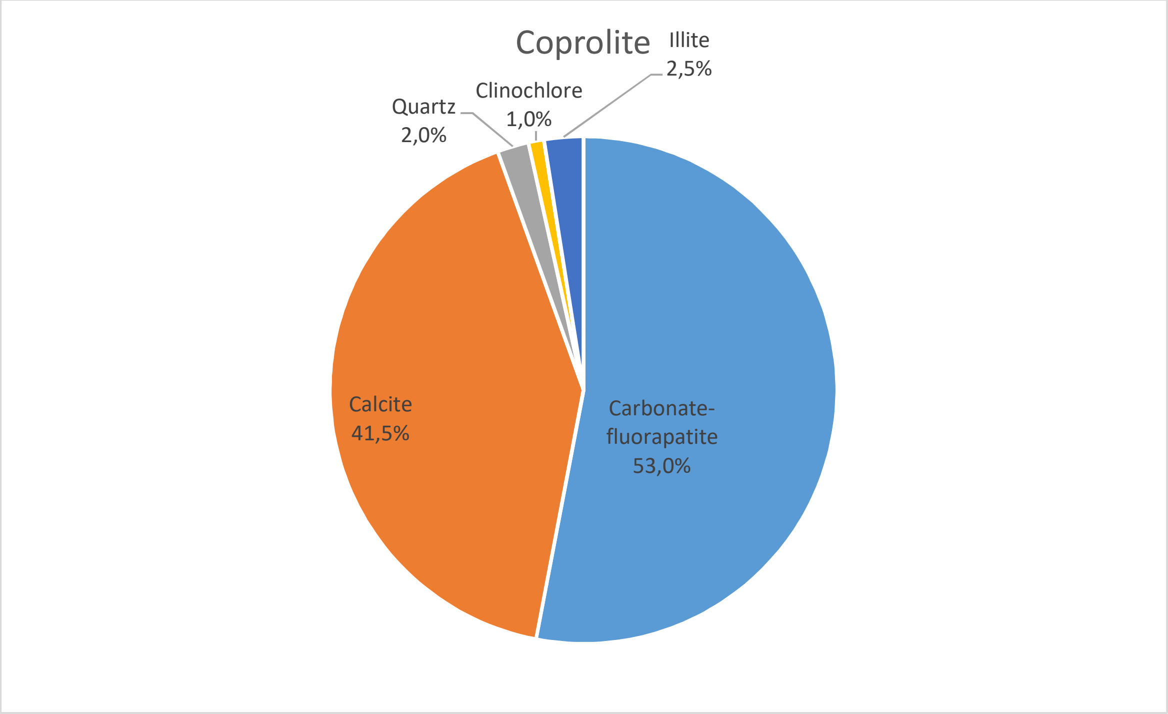
Coprolite
Illite 2,5%
Clinochlore 1,0%
Quartz 2,0%
Calcite 41,5%
Carbonate-fluorapatite 53,0%

verte!

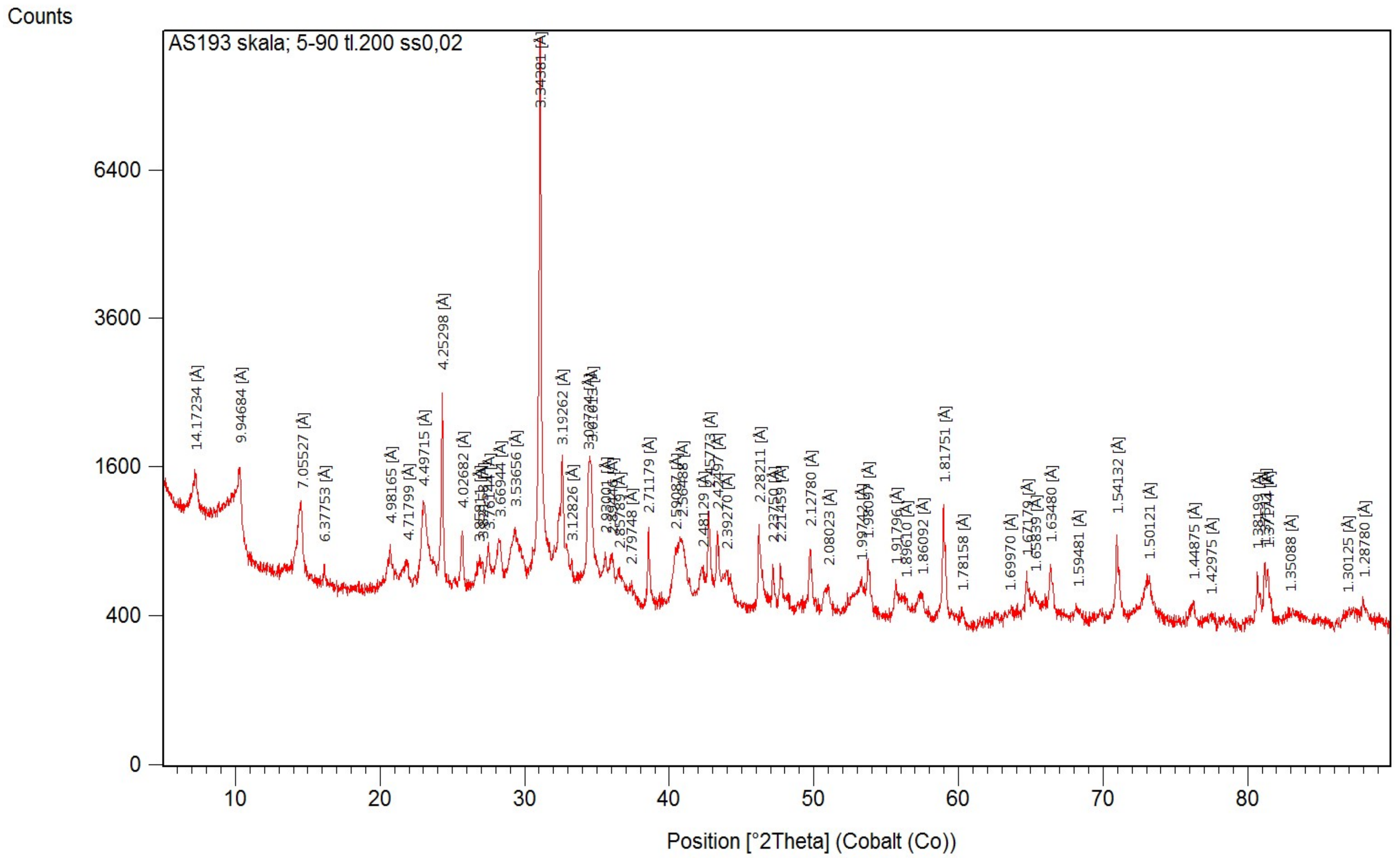
Counts
AS193 skala; 5-90 tl.200 ss0,02
6400
3600
1600
400
0
10
20
30
40
50
60
70
80
Position [°2Theta] (Cobalt (Co))
3.34381 [Å]
14.17234 [Å]
9.94684 [Å]
7.05527 [Å]
6.37753 [Å]
4.98165 [Å]
4.71799 [Å]
4.49715 [Å]
4.25298 [Å]
4.02682 [Å]
3.66944 [Å]
3.53656 [Å]
3.19262 [Å]
3.12826 [Å]
2.79748 [Å]
2.71179 [Å]
2.48129 [Å]
2.39270 [Å]
2.28211 [Å]
2.21459 [Å]
2.12780 [Å]
2.08023 [Å]
1.91796 [Å]
1.89610 [Å]
1.86092 [Å]
1.81751 [Å]
1.78158 [Å]
1.69970 [Å]
1.63480 [Å]
1.59481 [Å]
1.54132 [Å]
1.50121 [Å]
1.44875 [Å]
1.42975 [Å]
1.35088 [Å]
1.30125 [Å]
1.28780 [Å]

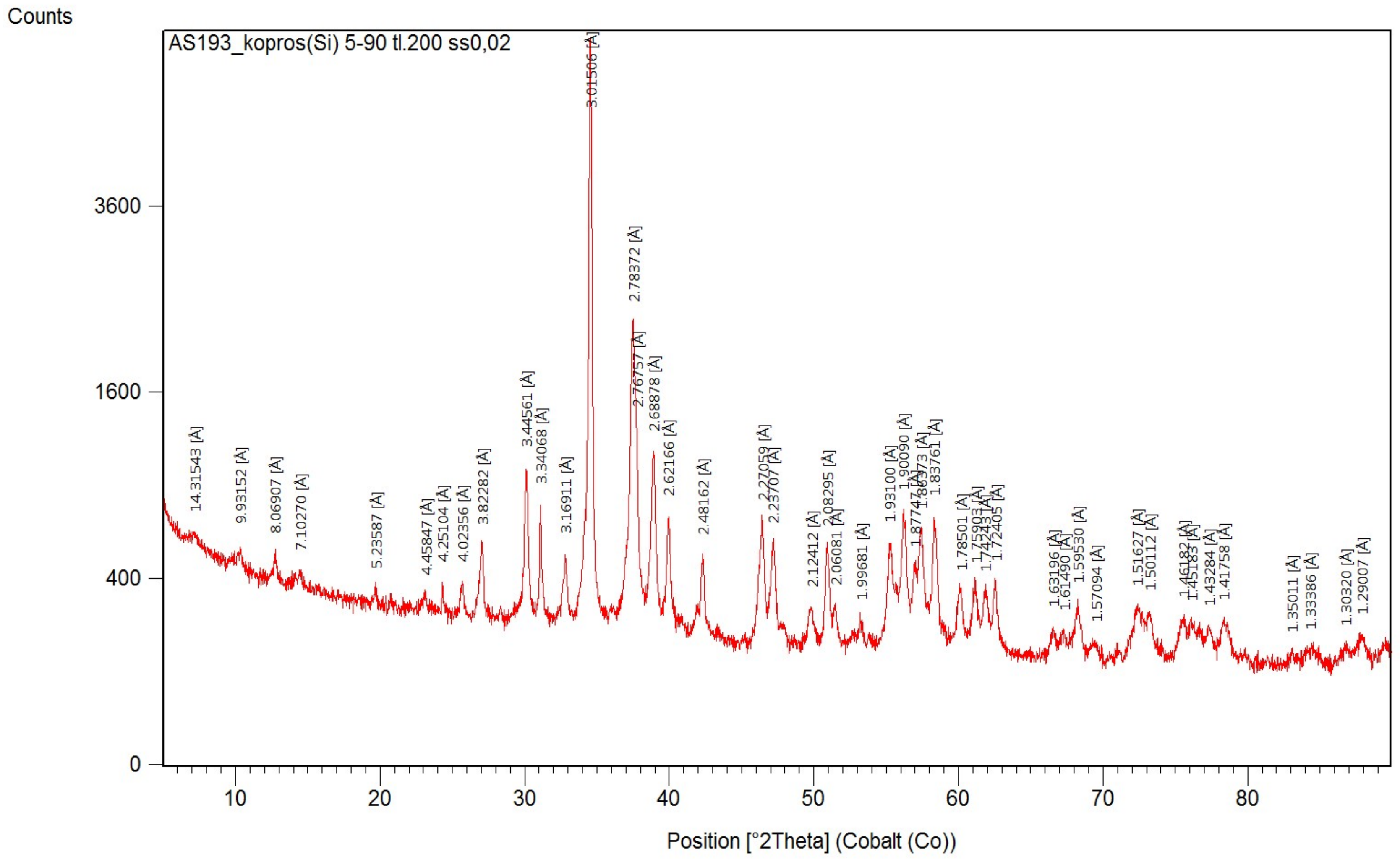
Counts
AS193_kopros(Si) 5-90 tl.200 ss0,02
3600
1600
400
0
10
20
30
40
50
60
70
80
Position [°2Theta] (Cobalt (Co))
14.31543 [Å]
9.93152 [Å]
8.06907 [Å]
7.10270 [Å]
5.23587 [Å]
4.45847 [Å]
4.25104 [Å]
4.02356 [Å]
3.82282 [Å]
3.44561 [Å]
3.34068 [Å]
3.16911 [Å]
3.01506 [Å]
2.78372 [Å]
2.76757 [Å]
2.68878 [Å]
2.62166 [Å]
2.48162 [Å]
2.27059 [Å]
2.23707 [Å]
2.12412 [Å]
2.08295 [Å]
2.06081 [Å]
1.99681 [Å]
1.93100 [Å]
1.90090 [Å]
1.83761 [Å]
1.78501 [Å]
1.72405 [Å]
1.63196 [Å]
1.61490 [Å]
1.59530 [Å]
1.57094 [Å]
1.51627 [Å]
1.50112 [Å]
1.43284 [Å]
1.41758 [Å]
1.35011 [Å]
1.33386 [Å]
1.30320 [Å]
1.29007 [Å]

Supplementary Figure S1. Raman spectrum for sample NHMW 2020/0033/0002 1910 A.

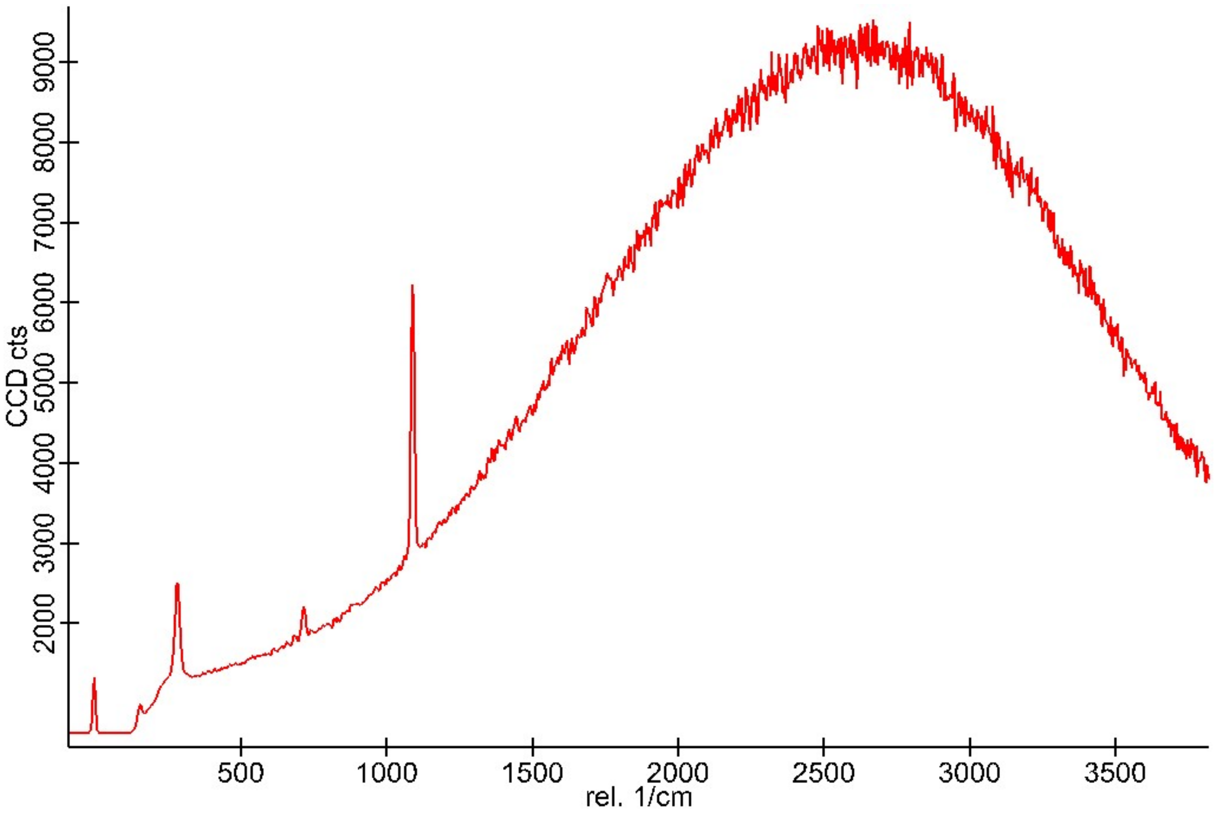

Supplementary Figure S2. XRD diffractograms for sample NHMW 2020/0033/0007 AS193.

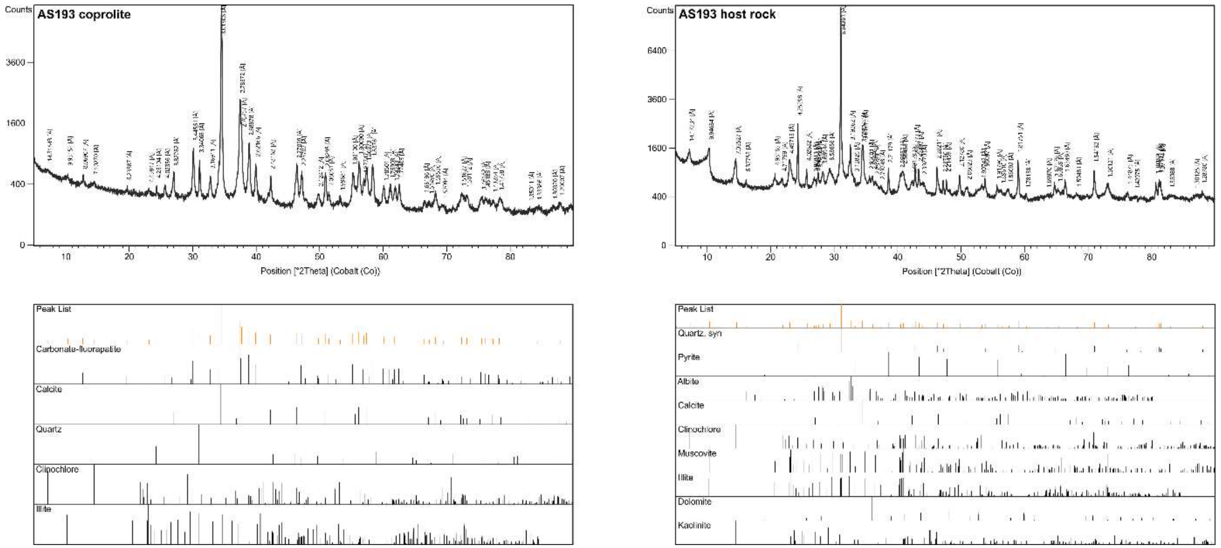

Supplement: Supplementary file 1 — Supplementary Information 1. [file 41598_2020_77017_MOESM1_ESM.pdf]
